# The locus coeruleus influences behavior by coordinating effective integration of fear memories and sensory input

**DOI:** 10.1371/journal.pbio.3003272

**Published:** 2025-07-14

**Authors:** Haoyu Duan, Tianyu Wang, Xinyang Zhang, Dan Xia, Zeyi Wang, Tsz Hei Fong, Tianxiang Li, Rongzhen Yan, Yang Zhan, Yulong Li, Wen-Jun Gao, Qiang Zhou

**Affiliations:** 1 State Key Laboratory of Chemical Oncogenomics, Guangdong Provincial Key Laboratory of Chemical Genomics, Peking University Shenzhen Graduate School, Shenzhen, China; 2 Cellular and Molecular Diagnostics Center, Sun Yat-sen Memorial Hospital, Sun Yat-sen University, Guangzhou, China; 3 Brain Cognition and Brain Disease Institute, Shenzhen Institutes of Advanced Technology, Chinese Academy of Sciences, Shenzhen, China; 4 State Key Laboratory of Membrane Biology, Peking University School of Life Sciences, Peking-Tsinghua Center for Life Science, IDG/McGovern Institute for Brain Research at PKU, Beijing, China; 5 Department of Neurobiology and Anatomy, Drexel University College of Medicine, Philadelphia, Pennsylvania, United States of America; Center for Brain Research, Medical University of Vienna, AUSTRIA

## Abstract

An essential function of memory is to guide behavior for better survival and adaptation. While memory formation has been extensively studied, far less is understood about how memory retrieval influences behaviors. In the auditory Pavlovian threat conditioning paradigm using C57BL/6J mice, retrieving a conditioned threat memory is associated with spiking in two dorsomedial prefrontal cortex (dmPFC) neurons with transient (T-neurons) and sustained (S-neurons) patterns. We show here that T-neurons and S-neurons are two distinct neuronal populations with different neuronal and synaptic properties and mRNA profiles. S-neuron spiking matches freezing behavior and is required for freezing. This sustained activity in S-neurons requires auditory inputs and the release of norepinephrine (NE) in the dmPFC. The activation of the locus coeruleus (LC) is initiated by dmPFC T-neuron inputs, sustained by auditory inputs, and is required for the transition to freezing by enhancing S-neuron activity. Interestingly, LC activation precipitates a brief period during which nonconditioned cues also induce freezing. Our findings highlight the critical contribution of the LC/NE system in the transition from memory to behavior, which coordinates the effective integration of memory, sensory inputs and emotional state for optimal adaptation.

## Author summary

This study provides new insights into the neural circuitry and mechanisms by which retrieved memories direct behaviors observed in the auditory threat conditioning model. Successful transition required interactions between various neuron types in the dmPFC and with LC neurons. These interactions determined and modulated the duration and intensity of defensive responses. Additionally, this study revealed that activating LC neurons can generalize behavior to nonconditioned cues, which may provide important insights for understanding certain anxiety disorders. These findings highlight the critical requirement of the LC/NE system as a neuromodulator in the transition to behavior and suggest that the LC/NE system may contribute to the emotional aspect of threat memory, which is widely regarded as emotional memory.

## Abstract 3

The neural circuits through which memory retrieval influences behaviors are not well understood. This study shows how fear memory activation initiates and sustains freezing behavior in mice through the interplay of norepinephrine and activation of different prefrontal neuron subtypes.

## Introduction

Understanding how memory translates to behavior is a key question in neuroscience. The formation of memories enables an adaptive association of predictive cues with relevant behavioral responses, whereas memory retrieval allows an organism to adopt an appropriate behavior guided by the learned cue to increase its probability of survival and adaptation [1–4]. While much has been revealed about the mechanisms underlying memory formation, much less is known about how the retrieved memories drive behavior. A deeper understanding of this process is crucial for understanding how memory influences or modulates behavior.

To address this question, a simple, well-defined model system with a robust behavior outcome is essential. An in-depth and systematic analysis of the circuitry and neurons participating in the process from memory retrieval to behavior is necessary to identify the key players and mechanisms to depict a general picture of this transition. In auditory Pavlovian or classical conditioning, coincident occurrence of a conditioned stimulus (CS, sound) and an unconditioned stimulus (US, footshock) leads to an association between the CS and US, which is a threat memory stored in the amygdala [4–8]. Re-encountering the same CS activates memory and a range of behavior, with freezing being the most commonly measured. Freezing is a defensive response consisting of a cessation of movement and a state of immobility. Retrieval of auditory threat memories initiates a cascade of events, including the activation of neurons in the lateral amygdala (LA), dorsomedial prefrontal cortex (dmPFC), basolateral amygdala (BLA), central nucleus of the amygdala (CeA), and periaqueductal gray (PAG), and ultimately driving the expression of freezing behaviors [5,7–11]. In this study, we used the above circuitry as a model system to examine the transition from memory to behavior.

During retrieval of auditory threat memory with a long CS (a few to dozens of seconds), two subsets of dmPFC neurons are activated: one with transient spiking (approximately 1 s; we termed them T-neurons) [12–15] and another with sustained spiking that roughly matches the CS duration (termed S-neurons) [16,17]. Since LA neurons exhibit transient responses to the CS [18,19] and are known to project to the PFC as part of the threat memory circuitry, it is likely that T-neurons receive LA inputs. Sustained responses in dmPFC S-neurons, which match the duration of CS and the duration of freezing, may project to the BLA since the PFC-BLA-PAG pathway is known to be an integral part of the freezing response activated by threat memory [9,16,20]. The matching of the durations of the CS and S-neuron responses suggests that these neurons may receive auditory inputs, which has not yet been directly tested. T-neurons and S-neurons are likely two distinct neuronal populations, but this distinction has not been shown directly. On the basis of these observations, we hypothesized that (1) dmPFC T- and S-neurons are two distinct neuronal populations with different inputs and outputs, with T-neurons receiving LA inputs whereas S-neurons receiving inputs from T-neurons and auditory areas and projecting to the BLA; (2) T-neurons project to S-neurons to propagate the retrieved threat memory to elicit freezing (behavioral) expression; (3) T- and S-neurons occupying nonoverlapping locations in the dmPFC but are likely in close proximity to each other; and (4) T- and S-neurons have distinct gene expression profiles and electrophysiological and pharmacological properties. In this study, we aimed to explore these hypotheses using a combination of tracing, *in vivo* and *in vitro* recording, pharmacology, optogenetic stimulation and manipulation, and RNA-seq analysis.

Another critical aspect in the retrieval of threat memory is a key contribution of NE. The contributions of NE to threat conditioning and extinction of memory have been extensively studied, but far less is known about its role in the expression of formed threat memories [21–23]. Elevated NE levels in the BLA during conditioning are required for memory formation [21,24,25]. During the retrieval of threat memory, NE levels are elevated in the PFC [26]. S-neuron responses are modulated by NE, and NE sustains CS-elicited PFC neuronal spiking and freezing [16,17,27]. These findings point to a much less explored area in the transition from memory to behavior via neuromodulators, particularly in terms of the underlying mechanism and functional importance. Key questions that need to be addressed include whether elevated NE levels are caused by locus coeruleus (LC) inputs to the PFC, and which inputs are activated by the CS and, in turn, activate the LC. Retrieved threat memory activates PFC neurons, and limited evidence suggests that projections from the PFC to LC/NE neurons occur. Therefore, the PFC may be a source of input to LC neurons during memory retrieval. A third question to be addressed is the mechanism by which higher NE levels affect dmPFC S-neuron responses. One possibility is that NE acts on β-receptors on S-neurons [16,17]. The NE is very likely associated with emotion-related processes. Although threat/fear memory is often regarded as emotional memory, the exact nature of this emotional aspect is not well defined or understood. High NE levels are associated with vigilance, arousal, stress and anxiety [28–33], and the LC/NE system is generally associated with attention, arousal and stress [34–38]. Hence, if NE mediates the emotional aspect of threat memory, it remains unclear whether NE possess some of the key properties of emotions, such as valence, scalability and generalization [39].

Here, we demonstrate that memory-activated dmPFC neurons and auditory inputs activate LC neurons that project back to the dmPFC and that the released NE enhances dmPFC S-neuron responses and enables freezing behavior. Interestingly, once activated, responses in the LC-neurons can be sustained by non-threat auditory cues, opening a brief window of generalization. Thus, a successful transition from memory to behavior requires the activation of stored memory, sensory inputs, and concomitant activation of LC neurons.

## Materials and methods

### Animals

Male C57BL/6J were purchased from Guangdong Medical Laboratory Animal Center (China). They were group housed (5–6 mice/cage) under a 12 hr light/dark cycle (8:00 AM to 8:00 PM) and were provided with food and water *ad libitum*. Bedding, water, and food were changed every week. Mice of 3–5 months of age were used. For *in vivo* electrophysiology experiments, mice were singly housed before the surgery to implant multi-wire electrodes. They were habituated by gentle handling for 3 days before behavioral and recording experiments. All animal experiments were performed in accordance with the ARRIVE (Animal Research: Reporting of *In Vivo* Experiments) guidelines, approved (Approval Number: 11530) by the Peking University Shenzhen Graduate School Animal Care and Use Committee.

### *In vivo* recording

Mice were anesthetized with isoflurane (induction 3%, maintenance 1.5%). Two screws were implanted to secure electrode array implants. Electrodes consisted of 16 individually insulated nichrome wires (35 μm inner diameter, impedance 300–900 KΩ; Stablohm 675, California Fine Wire) arranged in a 3 × 5 × 5 × 3 pattern (approximately 200 μm spacing between wires). Electrodes were attached to an 18-pin connector (Mil-Max) and secured with dental cement. Optrode (opto-electrode) arrays are composed of an optic fiber (250 μm) and multiwire electrodes (the end of the optical fiber is approximately 200–300 μm above the tips of the recording electrodes).

After surgery, mice were allowed to recover for 7–14 days. Broadband (0.3 Hz to 7.5 kHz) neural signals were simultaneously recorded (16 bits at 30 kHz) from implanted 16-channel arrays using a 64-channel data acquisition system (Zeus, Bio-Signal Technologies). At completion, recorded extracellular spikes were aligned and sorted (offline sorter software, Plexon), and further analyzed using NeuroExplorer (Nex Technologies) and MATLAB [14]. Responses during CS were normalized to pre-tone spike rate by calculating a *Ζ*-score [14]. For statistical analysis, comparisons were performed using the averaged Ζ-score values calculated during the 500 ms period after CS onset.

Neurons were considered CS responsive if they showed robust, time-locked changes in their spiking upon CS presentation. Neuron spiking within −1–0 s as an average basal level, when spiking above the average level adds 3 × S.E.M will be used as CS responsive.

The following are the coordinate positions of the brain regions involved in this experiment: dmPFC (1.98 mm anterior to Bregma; ±0.3 mm lateral to midline, and 2.15 mm below the Bregma plane); LA (1.75 mm posterior to Bregma; ±3.3 mm lateral to midline and 4.65 mm below the Bregma plane); BLA (1.31 mm posterior to Bregma, ±3.05 mm lateral to midline and 4.85 mm below the Bregma plane); LC (5.4 mm posterior to Bregma; ±0.85 mm lateral to midline and 4.5 mm below the Bregma plane); temporal association cortex (TeA, 3.52 mm posterior to Bregma; ±4.15 mm lateral to midline and 4.1 mm below the Bregma plane). These coordinates were used for the implantation of multiwire electrodes, injection of viruses, and placement of optical/imaging fibers. Notably, the target sites for optical fiber implantation were adjusted to be 0.1 mm above the specified coordinates.

### *In vivo* imaging

To record Ca^2+^ responses in the dmPFC neurons receiving LA inputs, mice were injected with the rAAV2/9-Ef1α-DIO-GCaMP7s virus in dmPFC, and rAAV1-CaMKII-Cre virus in LA. To record Ca^2+^ responses in the dmPFC neurons projecting to BLA, mice were injected with the rAAV2/9-Ef1α-DIO-GCaMP7s virus in dmPFC, and rAAV2/retro-hSyn-Cre virus in BLA. To record Ca^2+^ responses in the LC neurons, mice were injected with the rAAV2/9-CaMKII-GCaMP7s virus in LC. To record Ca^2+^ responses in the LC neurons (receiving input from or projecting to dmPFC), mice were injected with the rAAV2/9-Ef1α-DIO-GCaMP7s virus in LC, and rAAV1-CaMKII-Cre or rAAV2/retro-hSyn-Cre viruses in dmPFC. To record NE responses in the dmPFC and BLA, mice were injected with the rAAV-hSyn-NE2h or rAAV-hSyn-NE2m viruses in dmPFC and BLA. To record Ca^2+^ responses in the LC NE-neurons, mice were injected with the rAAV2/9-TH-GCaMP6s virus in LC.

Following virus injection, an optical fiber (230 μm O.D., 1.25 mm Ferrule Size) (Inper LLC) was placed in a ceramic ferrule and inserted towards the BLA, dmPFC or LC through a craniotomy. The ceramic ferrule was supported with a skull-penetrating M1 screw and dental acrylic. Fluorescence signal of GCaMP/NE2h was recorded with a multichannel fiber photometry system (Nanjing Thinker Tech, China). Excitation light from a 470 nm LED was reflected via a dichroic mirror (MD498; Thorlabs) and focused through a 20×/0.4 NA objective lens. An optical fiber cable (230 μm O.D., NA = 0.37, 2 m long) transmitted light from the objective through the implanted optical fiber. The laser power was adjusted at the tip of optical fiber to 20–25 μW to minimize bleaching. The GCaMP/NE2h fluorescence was band-pass filtered (MF525-39, Thorlabs) and collected using CMOS (DCC3240M, Thorlabs). A commercial software (Nanjing Thinker Tech, China) was used to transform images into time-series fluorescence signals and data were stored on a disk. The sampling rate was set to 50 frames per second.

For NE uptake inhibitor, 10 mg/kg duloxetine (Macklin, dissolved in sterile 0.9% saline) was administered (i.p.) 30 min before behavioral tests.

Data were analyzed using MATLAB. Quantification of fluorescence values (*ΔF*/*F*) were calculated and expressed as (*F *− *F*0)/*F*0, where *F*0 is the baseline fluorescence averaged between −10 and −2 s before CS presentation. The magnitudes of responses were measured by calculating the area under curve (AUC) throughout the entire CS duration.

### Fluorescence tracing and histology

To label dmPFC neurons that receive LA inputs and project to BLA, mice were injected with rAAV1-CaMKII-Cre virus in LA, rAAV2/9-Ef1α-DIO-BFP in dmPFC and retrobeads (red) in BLA. To label dmPFC neurons receiving TeA inputs, mice were injected with retrobeads (Red) in dmPFC. To determine whether dmPFC neurons receive TeA inputs, mice were injected with retrobeads (Red) in dmPFC. To determine whether LC neurons receive dmPFC, TeA or LA/BLA inputs, mice were injected with retrobeads (Red) in LC. To label the LC neurons that receive dmPFC inputs and send their projections back to dmPFC or to LA/BLA, mice were injected with the rAAV1-CaMKII-Cre virus in dmPFC and rAAV2/9-Ef1α-DIO-mCherry virus in LC. To label the dmPFC neurons that receive LA inputs and project to LC, mice were injected with the rAAV1-CaMKII-Cre virus in LA, rAAV2/9-Ef1α-DIO-mCherry virus in dmPFC and CTB 488 in LC.

To confirm the efficiency of Cre^+^ virus expression, mice were injected with rAAV2/retro-hSyn-Cre-mCherry virus in BLA, and rAAV2/9-hSyn-EGFP virus in dmPFC (S1A Fig). For Cre-DIO co-transduction assessment, mice were injected with rAAV2/retro-hSyn-Cre virus in BLA, rAAV2/9-hSyn-EGFP virus and rAAV2/9-Ef1α-DIO-mCherry virus in dmPFC (S1B Fig). Both Cre-dependent recombination (overlapping of EGFP^+^ and Cre-mCherry^+^ cells) and Cre-DIO co-transduction efficiency (overlapping of EGFP^+^ and DIO-mCherry^+^ cells) satisfied the experimental requirement (S1 Fig).

Histological procedures were performed. An overdose of sodium pentobarbital was used to anesthetize the mice, after which they were perfused through the heart with 1× PBS followed by a fixative solution containing 4% paraformaldehyde in PBS. Mouse brains were then removed and post-fixed in the aforementioned fixative solution at 4 °C overnight. The samples were subsequently transferred to a sucrose solution consisting of 30% sucrose in PBS and stored at 4 °C for 48 hr. Mouse brains were then embedded in O.C.T. compound (Optimal cutting temperature compound) and sectioned coronally at a thickness of 30 μm using a cryostat at −20 °C. Sections containing the regions of interest were collected and preserved. Coronal sections with a thickness of 30 μm were analyzed to confirm the expression of the virus in the brain.

### Slice recording and optogenetic stimulation

Mouse brains were rapidly removed and placed in an ice-cold cutting solution containing (in mM): 110 choline chloride, 7 MgSO_4_, 2.5 KCl, 1.25 NaH_2_PO_4_, 25 NaHCO_3_, 25 D-glucose, 11.6 sodium ascorbate, 3.1 sodium pyruvate, and 0.5 CaCl_2_ gassed with 95% O_2_ and 5% CO_2_. Slices of 350 μm thickness were cut with a VT-1200S Vibratome tissue slicer (Leica Biosystems, Germany) in cutting solution. Slices were transferred to a holding chamber with artificial cerebrospinal fluid ACSF containing (in mM): 127 NaCl, 2.5 KCl, 1.25 NaH_2_PO_4_, 25 NaHCO_3_, 25 D-glucose, 2 CaCl_2_, and 1 MgSO_4_, and allowed to recover for 30 min at 32 °C, then kept at room temperature for at least 1 hr before recording. Individual slices were transferred to the recording chamber on an Olympus microscope (BX51WI) with a ×40 water-immersion differential interference contrast objective. Slices were constantly perfused at room temperature (23–26 °C) with oxygenated aCSF (4–5 mL/min). Recordings were made from layer 2/3 of the dmPFC, in a depth of about 50–100 μm in the slices.

Data were acquired using HEKA EPC10 double patch clamp amplifier (HEKA). Signals were acquired at a sampling rate of 10 kHz and filtered at 2 kHz. Series resistance of the recording pipette was between 10 and 25 MΩ. Neurons with holding current > −200 pA (at −70 mV) were excluded from data analysis. To record spontaneous excitatory post-synaptic currents (sEPSCs) in T- and S-neurons, somatic whole-cell voltage clamp recordings (−70 mV) were obtained from layer II/III excitatory neurons in dmPFC. Recording electrodes were filled with (in mM): 125 CsMeSO4, 5 NaCl, 1.1 EGTA, 10 HEPES, 0.3 Na_2_GTP, 4 Mg-ATP, and 5 QX-314. To record spontaneous inhibitory post-synaptic currents (sIPSCs), somatic whole-cell voltage clamp recordings (+5 mV) were obtained from layer II/III excitatory neurons in the dmPFC. Current clamp recordings were used to examine spikes evoked by a series of 500 ms depolarizing current pulses with 4 s intervals, and each injection step with an increase of 25 pA (from 0 to 375 pA). Recording pipette solution contains (in mM): 128 K+-gluconate, 10 NaCl, 2 MgCl_2_, 10 Hepes, 0.5 EGTA, 4 Na_2_ATP, and 0.4 NaGTP. All neurons were recorded for at least 5 min. Resting membrane potentials (RMPs) were measured at *I* = 0 pA, and neurons with RMPs above −50 mV were excluded from the analysis. The sEPSCs were recorded in the LC neurons in response to stimulation of the TeA axonal terminals (ChrimsonR), or combined opto-stimulation of PFC axonal terminals (ChR2) and TeA axonal terminals, at a holding membrane potential of −70 mV.

To identify T-neurons and S-neurons in brain slice, we injected rAAV1-CaMKII-Cre anterior transport virus in the LA and rAAV2/9-EF1α-DIO-ChR2-EGFP virus in the dmPFC, and injected retrobeads (red) in BLA. The dmPFC T-neurons showed EGFP fluorescence (green) and S-neurons showed retrobeads fluorescence (red). To activate TeA/dmPFC axonal terminals in LC neurons, we injected rAAV2/9-CaMKII-ChrimsonR-mCherry virus in TeA and rAAV2/9-CaMKII-ChR2-mCherry virus in dmPFC. To activate dmPFC axonal terminals in LC neurons (projecting to dmPFC), we injected rAAV2/9-CaMKII-ChR2-mCherry virus and retrobeads (green) in the dmPFC. All optogenetic stimulations were conducted with the same parameters based on the specific opsin used: a 473-nm laser (20 ms pulse width, 20 Hz, approximately 12 mW) for ChR2 activation whereas a 564-nm laser (20 ms pulse width, 20 Hz, approximately 12 mW) for ChrimsonR activation.

### Behavioral assays

Threat conditioning and retrieval test were performed in two contexts (threat conditioning in context A; retrieval test in context B). Context A and context B were different in their shapes and odors. Chamber floors were cleaned with 1% acetic acid or 75% ethanol before testing. Scoring freezing behavior was done using a video recording system (Coulbourn Instruments). Mice were considered freezing if no movement was detected for at least 2 s. On day 0, mice were habituated in context A and received 4 CS (conditioned stimulus; 30 s, 60 dB, 50 ms pips tone, 3 kHz). Threat conditioning was conducted on day 1, with CS co-terminated with a US (unconditioned stimulus; 2 s footshock, 0.75 mA), for 3 CS-US pairing trials. Training trials were separated by 90 s intervals. For threat memory retrieval on day 2, conditioned mice received 4 trials of various combinations of CS+ and CS− (30 s, 60 dB; CS+: 50 ms pips tone (3 kHz); CS−: white noise) in context B. For experiments with Calcium/NE signal imaging, conditioned mice received 3 trials of 30 s CS+, 10 s CS+, 2 s CS+ or 2 s CS+/28 s CS− in context B at 24 hr post-conditioning.

The selection of CS+ durations (30, 10, 2, and 2 s CS+/28 s CS−) were based on the following rationale: 30 s CS+ and 10 s CS+ were used to examine whether calcium/NE signals were temporally matched to CS duration; 2 s CS+ was used to mimic the activation of T-neurons, whereas 2 s CS+/28 s CS− was used to probe post-activation effects following T-neurons.

### *In vivo* optogenetic manipulations

Mice were injected with rAAV2/9-CaMKII-eNpHR3.0-mCherry virus or rAAV2/9-CaMKII-ChR2-EGFP virus bilaterally in LA and opto-fiber was placed in the dmPFC to inhibit or activate LA axonal terminals in dmPFC. Mice were injected with rAAV2/retro-hSyn-Cre virus bilaterally in the BLA, rAAV2/9-Ef1α-DIO-eNpHR3.0-mCherry virus in dmPFC, and opto-fiber was placed in the dmPFC to inhibit BLA-projecting dmPFC neurons [40,41]. Mice were injected with rAAV2/retro-hSyn-Cre virus in dmPFC, rAAV2/9-Ef1α-DIO-eNpHR3.0-mCherry virus in LC and opto-fiber was placed in the LC to inhibit dmPFC-projecting LC neurons. Mice were injected with rAAV1-CaMKII-Cre virus in dmPFC, rAAV2/9-Ef1α-DIO-ChR2-mCherry virus in the LC and optical fiber was placed in the LC to activate LC neurons (receiving dmPFC inputs). To activate TeA excitatory neurons, rAAV2/9-CaMKII-ChR2-mCherry virus was injected to TeA bilaterally, and opto-fiber was placed in the TeA. For optogenetic inhibition experiments involving eNpHR3.0, a 593-nm laser system was employed with continuous illumination (approximately 10 mW at the end of the optical fibers).

### *In vivo* pharmacogenetic manipulations

Mice were injected with the rAAV2/9-Ef1α-DIO-hM4D (Gi)-mCherry virus in the dmPFC and rAAV2/retro-hSyn-Cre virus in BLA to inhibit dmPFC neurons that project to BLA. Four weeks later, mice were injected intraperitoneally with CNO (3.3 mg/kg, MedChemExpress) 30 min before threat memory retrieval.

### Intra-cerebroventricular injection

Mice were anesthetized with isoflurane and placed in a stereotaxic apparatus. A guide cannula (2.5 mm in diameter) was implanted into the brain at coordinates relative to bregma: anteroposterior (AP) −0.20 mm, mediolateral (ML) −1.0 mm, and dorsoventral (DV) −2.0 mm. Following recovery, 5 μL of saline or 5 μL of propranolol (1 μg/μL, total 5 μg) was administered intracerebroventricularly (i.c.v.) through the cannula 30 min prior to the experiments [42].

### Cell harvesting

The T- and S-neurons were identified in brain slices using their selective fluorescence markers, respectively. Weak negative pressure was applied to aspirate the entire neuron into the glass patch electrodes, which were treated with DEPC water (ThermoFisher) in advance [43]. The pipette tip was broken onto the wall of a 0.2 mL tight-lock tube (TubeOne) to allow the entire neuron to be immersed in a 1 μL drop of RNase-free lysis buffer (provided by Beijing Genomics Institution (BGI)) placed on the side of the tube. The tube was kept on ice and two neurons were collected in one tube within 5 min. The tube was then placed in dry ice until 20 neurons were harvested from each mouse. Samples were rapidly spun down (5–10 s) and stored at −80 °C before reverse transcription. Reverse transcription, PCR amplification and sequencing were performed by BGI.

### RNA sequencing

A total of 6 samples were sequenced separately and were performed as before [43]. The expression amount of identified RNAs was analyzed using TPM (transcripts per million). Differential expression analysis was conducted using DESeq2 package in R software (1.2.22). Differentially expressed genes (DEGs) were defined as those with |fold change| ≥ 2 and *q*-value (a corrected *P*-value using Benjamini and Hochberg multiple testing correction) <0.05.

### Statistical analysis and graphing

RNA Sequencing figures were generated using Dr. Tom online system and Adobe Illustrator CC 2018. Other figures were generated by GraphPad Prism. Statistical significance was calculated using a two-tailed paired/unpaired *t* test, one-way/two-way repeated measures (RM) ANOVA (GraphPad Prism), as noted. Data are reported as mean ± SEM. Significance levels are noted as * *p* < 0.05; ** *p* < 0.01; *** *p* < 0.001.

## Results

### Conditioned threat cues induce transient and sustained neuronal responses in the dmPFC

**Fig 1:**
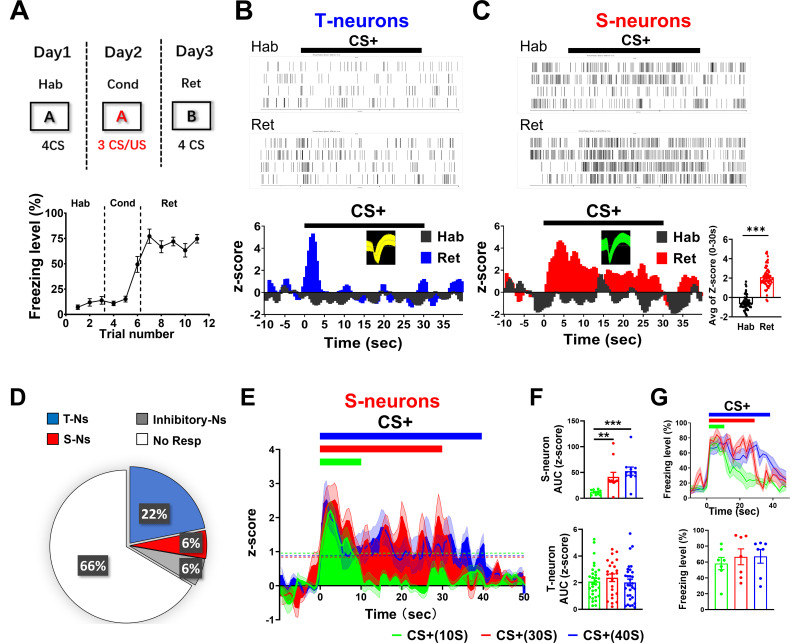
Activity of dmPFC T-neurons and S-neurons during retrieval of threat memory. **(A)** (Upper) Experimental procedure for threat conditioning. (Lower) Freezing levels before, during, and after auditory threat conditioning. *N* = 12 mice. **(B)** (Upper) Raster plots of spiking in representative T-neurons during habituation (Hab) and CS retrieval after conditioning (Ret). (Lower) Peri-event histograms showing spiking in the T-neurons. Bars indicate CS presentation. Insert: spike waveform. Bin width, 0.5 s. *n* = 23 units/5 mice. **(C)** (Upper) Raster plots of spiking in representative S-neurons during habituation and CS retrieval after conditioning. (Lower left) Peri-event histograms of S-neuron spiking. (Lower right) Averaged *z*-scores vales for S-neurons during habituation and retrieval. Insert: spike waveform and Avg of *Z*-score (0–30 s). Two-tailed unpaired *t* test, Hab vs. Ret, *P* < 0.001. *n* = 17 units/5 mice. **(D)** Distribution of recorded dmPFC neurons based on their responses to CS in conditioned mice. About 22% (151/692) showed a transient increase, 6% (41/692) sustained increase, 66% (460/692) no response and 6% (40/692) were inhibitory neurons and. *n* = 692 units/42 mice. **(E)** Spiking in S-neurons elicited by three CSs with different durations. Dotted lines indicate *z*-score = 1. **(F)** (Upper) The AUC (*z*-score) of CS-elicited S-neuron response. CSs correspond to those used in **(E)**. One-way RM ANOVA, *F* (2, 45) = 9.671, Bonferroni’s posttest; CS+ (10 s) vs. CS+ (30 s), *P* < 0.01; CS+ (10 s) vs. CS+ (40 s), *P* < 0.001; *n* = 16 units/8 mice. (Lower) The AUC (*z*-score) of CS-elicited responses in T-neurons (*n* = 29 units/8 mice). **(G)** (Upper) Freezing levels and time courses elicited by three CSs. One-way RM ANOVA, *F* (2, 18) = 15.98, Bonferroni’s posttest; CS+ (10 s) vs. CS+ (30 s), *P* < 0.01; CS+ (10 s) vs. CS+ (40 s), *P* < 0.001. (Lower) Freezing levels elicited by three CSs. *N* = 7 mice. Unless specified, statistical comparisons were performed using two-tailed unpaired *t* test; *, *P* < 0.05; **, *P* < 0.01; ***, *P* < 0.001. Data represented as mean ± SEM. Numerical da*t*a can be found in S1 Data.

We simultaneously examined spiking in dmPFC neurons and freezing levels in mice [14,15,44]. Although both transient and sustained neuronal responses have been shown in prelimbic/dmPFC neurons in response to CS+ (conditioned CS, paired with US) after auditory threat conditioning, they have not been demonstrated in the same mice. We first recorded dmPFC neuronal responses to CS presentation in conditioned mice (Fig 1A–1C). CS+ elicited two spiking patterns in two nonoverlapping neuronal populations recorded in the dmPFC in the same mice, with either transient (T-neurons) (Fig 1B) or sustained (S-neurons) responses (Fig 1C). Consistent with previous reports, T-neuron responses lasted approximately 1 s (Fig 1B), whereas S-neuron responses roughly matched the CS+ duration (Fig 1C). Both increased and decreased sustained responses were observed, but only increased transient responses were observed (S2 Fig). No obvious responses to CS− were observed, indicating the selectivity of these responses to the conditioned stimulus (S2A Fig). Among the 692 dmPFC neurons recorded, 22% (151/692) exhibited transient responses, while 6% (41/692) exhibited sustained responses, and 6% (40/692) were inhibitory neurons with increased responses (based on their spike waveforms, S2C Fig) (Fig 1D). The remaining recorded dmPFC neurons did not display obvious CS-induced changes in spiking. The ratio of total T-neurons to S-neurons was 3.68. For the remainder of the study, we present only the results from the dmPFC excitatory neurons that responded to the CS.

In our experiments, a 30 s long CS+ was used during both the conditioning and retrieval tests. To determine whether the durations of the conditioned freezing responses and S-neuron spiking match those of the CSs with different durations, we used three different CS+ durations (10, 30 and 40 s). We observed that the durations of S-neuron spiking were well matched with those of the corresponding CSs (Fig 1E, 1F), whereas T-neurons responded with similar amplitudes and durations regardless of the duration of CS+ (Fig 1F). In addition, freezing durations matched the CS+ durations, whereas freezing levels were comparable (Fig 1G). In comparison, the majority of LA excitatory neurons only showed transient spiking to CS+ (S2B Fig) [18,19,45]. In summary, transient and sustained responses were observed in two nonoverlapping dmPFC neuron populations with distinct response profiles, suggesting differential contributions to threat memory.

### Distinct connection patterns of dmPFC T-neurons and S-neurons

**Fig 2:**
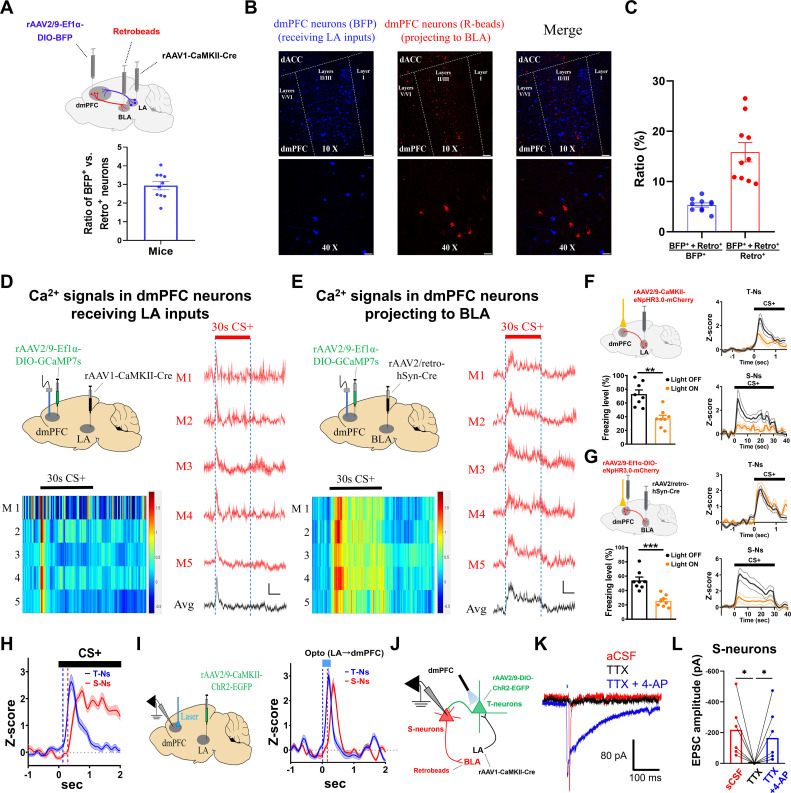
Distinct responses in dmPFC T-neurons and S-neurons, their inputs, outputs and connections with each other. **(A)** (Top) Diagram showing the virus injection sites. The dmPFC neurons receiving LA inputs were labeled with BFP, BLA-projecting dmPFC neurons were labeled with retrobeads (red). (Bottom) Ratio of BFP^+^ vs. Retro^+^ neurons (each dot represents averaged values from a single mouse). *N* = 47 sections/10 mice. **(B)** Representative images showing the spatial localization of labeled dmPFC neurons with BFP (blue) or retrobeads (red), taken using 10 X (upper) or 40 X (lower) objectives. The majority of labelled neurons were located in layer II/III of dmPFC. Scale bars, 100 μm (10 X) and 25 μm (40 X). **(C)** Numbers of neurons positive for both BFP^+^ and Retro^+^ over BFP^+^ neurons (blue) and numbers of neurons positive for both BFP^+^ and Retro^+^ over Retro^+^ neurons (red). *N* = 10 mice. **(D)** (Left, top) Sites for virus injections and Ca^2+^ response recording; (Left, bottom), Heat maps showing Ca^2+^ signals elicited by 30 s CS+ (dotted lines) in dmPFC neurons receiving LA inputs. (Right) Averaged Ca^2+^ signals from individual mouse (red traces) and the averaged responses from all mice (black), elicited by 30 s CS+ in dmPFC neurons receiving LA inputs. Scale bars, 1% *ΔF*/*F* and 10 s. *N* = 5 mice. **(E)** (Left, top) Sites for virus injections and Ca^2+^ response recording; (Left, bottom) Heat maps showing Ca^2+^ signals elicited by 30 s CS+ in dmPFC neurons projecting to BLA. (Right) Averaged Ca^2+^ signals from individual mouse (red traces) and averaged responses from all mice (black), elicited by 30 s CS+ in dmPFC neurons projecting to BLA. Scale bars, 1% *ΔF*/*F* and 10 s. *N* = 5 mice. **(F)** (Left, top) Sites of virus injections and optogenetic inhibition; (Left, bottom) Opto-inhibition of LA axonal terminals in the dmPFC on freezing levels during CS+ retrieval. Two-tailed paired *t* test, Laser ON vs. Laser OFF, *P* < 0.01; *N* = 8 mice. (Right) Opto-inhibition of LA axonal terminals in dmPFC on spike rates in T-neurons (top) and S-neurons (bottom), during CS+ retrieval. T-neurons, *n* = 35 units/8 mice; S-neurons, *n* = 20 units/8 mice. **(G)** (Left, top) Sites of virus injections and optogenetic inhibition; (Left, bottom) Opto-inhibition of BLA-projecting dmPFC neurons on freezing levels during CS+ retrieval. Two-tailed paired *t t*est, Light ON vs. Light OFF, *P* < 0.001; *N* = 8 mice. (Right) Opto-inhibition of BLA-projecting dmPFC neurons on spike rates in dmPFC T-neurons (top) and S-neurons (bottom), during CS+ retrieval. T-neurons, *n* = 41 units/8 mice; S-neurons, *n* = 24 units/8 mice. **(H)** Response latency of dmPFC T-neurons and S-neurons elicited by CS+. Dotted lines represent the time points when slope (*dy*/*dx*) of responses were equal to 1 (points of intersection were 0.122 and 0.298 s, respectively). T-neurons, *n* = 54 units/12 mice; S-neurons, *n* = 29 units/12 mice. **(I)** (Left) Sites of virus injections and dmPFC recording. (Right) Responses in dmPFC T-neurons and S-neurons to opto-stimulation of LA neuron axonal terminals in dmPFC (bar). Dotted lines represented the time points when slope (*dy*/*dx*) of responses were equal to 1 (points of intersection were 0.008 and 0.159 s, respectively). T-neurons, *n* = 32 units/6 mice; S-neurons, *n* = 16 units/6 mice. **(J)** Schematic diagram showing opto-activation of T-neurons and recording in S-neurons in PFC slices. **(K)** Representative EPSC traces in the S-neurons (projecting to BLA) elicited by opto-stimulation of dmPFC neurons receiving LA inputs (5 ms, bar), in sequential presence of aCSF, TTX, and TTX + 4-AP. The EPSC trace during TTX bath application (black) was rightward shifted for better visualization. Scale bars, 100 ms and 80 pA. **(L)** EPSC amplitudes in the S-neurons from H_2_. *n* = 6 cells/3 mice, paired *t* tes*t*, *P* < 0.05. Numerical data can be found in S1 Data.

The above differences in the CS+-elicited responses in the dmPFC T- and S-neurons suggest that they may have distinct connection patterns. Previous studies have indicated that dmPFC neurons receive synaptic inputs from the LA [5,14,15,18] and that some dmPFC neurons project to the BLA [16,17]. To confirm these findings under our experimental conditions, we labeled the dmPFC neurons receiving LA inputs with BFP (BFP^+^, rAAV1-CaMKII-Cre virus injected in the LA and rAAV2/9-Ef1α-DIO-BFP virus in the dmPFC) and BLA-projecting dmPFC neurons with retrobeads (Retro^+^, injected in the BLA; Fig 2A). The majority of labeled neurons (BFP^+^, Retro^+^) were located in layers II/III of the dmPFC, with approximately 92.54% of LA projections terminating in layers 2/3 and 7.46% in layer 5 (Fig 2B). The ratio of BFP^+^ to Retro^+^ neurons was 2.94 (Fig 2A), which was consistent with the ratio of T-neurons to S-neurons from *in vivo* recordings. About 5.35% of BFP^+^ neurons and 15.84% of Retro^+^ neurons were positive for both BFP^+^ and Retro^+^, suggesting minimal overlap between these two neuronal populations (Fig 2C).

The above findings suggest that dmPFC T-neurons and S-neurons may be differentially tagged and studied. We first examined the CS+-elicited Ca^2+^ responses in dmPFC neurons receiving LA inputs by injecting rAAV1-CaMKII-Cre virus into the LA and rAAV2/9-Ef1α-DIO-GCaMP7s virus into the dmPFC (Fig 2D). These Ca^2+^ responses were clearly transient (approximately 1 s) (Fig 2D) and therefore indicate that dmPFC neurons receiving LA inputs respond to CS+ in a transient manner, similar to the response pattern of T-neurons from our *in vivo* recordings. We then examined Ca^2+^ responses in dmPFC neurons projecting to the BLA by injecting rAAV2/retro-hSyn-Cre virus into the BLA and rAAV2/9-Ef1α-DIO-GCaMP7s virus into the dmPFC (Fig 2E). These neurons showed sustained responses to CS+, roughly matching the CS+ duration (Fig 2E), similar to the S-neurons recorded *in vivo.* Taken together, these results indicate that the two dmPFC neuron populations show distinct responses to CS+ on the basis of their inputs or projections. This finding provides the foundation for further analysis of their properties and selective manipulation of their activities.

The implication of the above finding is that T-neurons and S-neurons can be selectively targeted to manipulate their activities. This manipulation will allow further investigation of their contributions to the generation of freezing behavior. To this end, we used opto-inhibition of the LA neuron axonal terminals in the dmPFC by expressing the rAAV2/9-CaMKII-eNpHR3.0-mCherry virus in the LA (Fig 2F). Compared with no-light conditions, yellow light illumination during CS+ led to reduced freezing levels and spiking in both T-neurons and S-neurons (Fig 2F). Opto-inhibition of BLA-projecting dmPFC neurons using retrograde virus (rAAV2/retro-hSyn-Cre virus in the BLA and rAAV2/9-Ef1α-DIO-eNpHR3.0-mCherry virus in the dmPFC) significantly reduced freezing levels (Fig 2G). Importantly, an obviously lower spiking rate was observed in the S-neurons but not in the T-neurons (Fig 2G).

The above findings indicate that T-neurons are upstream of S-neurons in the propagation of threat memory-related information from the LA to the BLA. Consistent with this, CS+-elicited spiking in the S-neurons lagged spiking in the T-neurons by approximately 200 ms (Fig 2H). To further test whether S-neurons receive inputs from T-neurons, we injected the rAAV2/9-CaMKII-ChR2-EGFP virus into the LA (Fig 2I). Opto-stimulation of the axonal terminals in the dmPFC resulted in spiking in the T-neurons followed by spiking in the S-neurons by approximately 200 ms (Fig 2I). Interestingly, opto-stimulation of the T-neurons evoked transient responses in the S-neurons, suggesting that the sustained spiking in the S-neurons is likely caused by the long CS.

As shown in both Fig 2H and 2I, we observed a 200 ms delay between spiking in T-neurons and S-neurons. One explanation for this long delay is that T-neuron projections onto S-neurons are polysynaptic. To investigate this possibility, we injected the rAAV1-CaMKII-Cre virus into the LA and the rAAV2/9-Ef1α-DIO-ChR2-EGFP virus into the dmPFC to activate T-neurons via anterograde transport and then injected the retrobeads (red) into the BLA to label S-neurons (Fig 2J). The dmPFC S-neurons were identified by fluorescence (Fig 2J). Light-evoked EPSCs were recorded in S-neurons, and these EPSCs were eliminated by bath perfusion with tetrodotoxin (TTX) but rescued by further addition of 4-aminopyridine (4-AP) in the perfusion medium (Fig 2K; 2L). Since monosynaptic but not polysynaptic connections are preserved in TTX + 4-AP [46], this result indicates that T-neurons project to S-neurons monosynaptically. The 200 ms delay in Fig 2H and 2I is likely caused by the amount of time required for synaptic integration to reach the spiking threshold.

### Distinct neuronal properties of T-neurons and S-neurons

**Fig 3:**
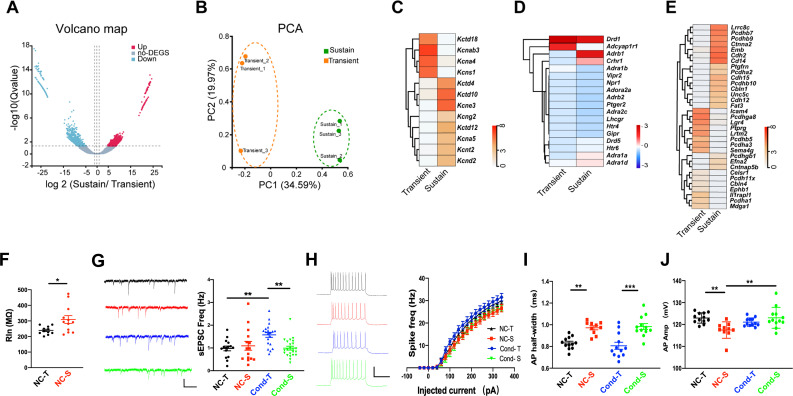
Characterizations of T-neurons and S-neurons. **(A)** Volcano plots showing significantly differentially expressed genes (DEGs) in the T-neurons and S-neurons. **(B)** Principal components analysis (PCA) of an RNA-seq data set of 6 samples was sufficient to distinguish between T-neurons and S-neurons. **(C)** Heat map showing average transcripts per million (TPM) of differentially expressed genes encoding potassium channels with significance. Heatmap was standardized by rows. **(D)** Heat map showing average TPM of differentially expressed genes related to norepinephrine signaling with significance. **(E)** Heat map showing average TPM of differentially expressed genes encoding cell adhesion molecules with significance. In all heatmaps, the redder the color, the higher the expression level. Standardized method: log (average TPM + 1). *n* = 20 cells/mouse, 3 mice each group. **(F)** Input resistance of T-neurons and S-neurons from non-conditioned (NC) mice. Two-tailed unpaired *t* test, NC-T vs. NC-S, *P* = 0.0179; *n* = 11 cells/3 mice (NC-T), 13 cells/3 mice (NC-S). **(G)** (Left) Representative sEPSC traces and (right) sEPSC frequency in dmPFC T-neurons and S-neurons, from conditioned (Cond) and non-conditioned (NC) mice. One-way RM ANOVA, *F* (2, 53) = 7.804, Bonferroni’s posttest, NC-T vs. Cond-T, *P* < 0.01; NC-S vs. Cond-S, *P* < 0.01; *n* = 15 cells/4 mice (NC-T), 15 cells/4 mice (NC-S), 20 cells/4 mice (Cond-T), 24 cells/6 mice (Cond-S). Scale bars, 20 pA and 500 ms. **(H)** (Left) Representative traces of action potentials in dmPFC T-neurons and S-neurons elicited by injection of current through the recording electrodes. (Right) Spike frequency of action potentials plotted against injected currents. Two-way RM ANOVA, *F* (30, 990) = 6.894, Bonferroni’s posttest, *P* < 0.001; NC-T vs. NC-S, *P* < 0.01; NC-T vs. Cond-T, *P* < 0.001; *n* = 10 cells/4 mice (NC-T), 11 cells/4 mice (NC-S), 11 cells/4 mice (Cond-T), 21 cells/6 mice (Cond-S). Scale bars, 50 mV and 200 ms. **(I)** Half-width of action potentials (AP) in the T-neurons and S-neurons. One-way RM ANOVA, *F* (3, 42) = 13.57, *P* < 0.001, Bonferroni’s posttest; NC-T vs. NC-S, *P* < 0.01; Cond-T vs. Cond-S, *P* < 0.001; *n* = 12 cells/4 mice (NC-T), 10 cells/4 mice (NC-S), 12 cells/4 mice (Cond-T), 12 cells/6 mice (Cond-S). **(J)** Amplitude of AP in the T-neurons and S-neurons. One-way RM ANOVA, *F* (3, 41) = 5.530, *P* < 0.01, Bonferroni’s posttest; NC-T vs. Cond-T, *P* < 0.05; *n* = 12 cells/4 mice (NC-T), 9 cells/4 mice (NC-S), 12 cells/4 mice (Cond-T), 12 cells/6 mice (Cond-S).

We next asked whether T-neurons and S-neurons possess different properties in addition to the distinct connection patterns. To answer this question, we first conducted an RNA-seq analysis. We aspirated RNAs from labeled T-neurons or S-neurons from brain slices using patch electrodes and examined their RNA profiles [43]. Here, T-neurons were defined as those dmPFC neurons receiving LA inputs, whereas S-neurons were defined as those dmPFC neurons projecting to the BLA. A total of 14,609 genes were detected (S2 Table), with 650 showing significantly higher expression and 728 with lower expression in the S-neurons than in the T-neurons (Fig 3A and S2 Table). Principal component analysis of the 6 samples distinguished between T-neurons and S-neurons (Fig 3B). We first found that for ion channels, the expression of 8 genes (*Kctd4, Kctd10, Kctd12, Kcne3, Kcng2, Kcna5, Kcnt2 and Kcnd2*) was significantly greater and that of 4 genes (*Kctd18, Kcnab3, Kcna4 and Kcns1*) was significantly lower in the S-neurons (Fig 3C and S3 Table), whereas the expression of *Cacnb2* was significantly greater in the S-neurons, with no difference in the expression of sodium channel genes (S3 Table). These results suggest differences in neuronal excitability between T- and S-neurons. Second, no difference was detected for GABA receptors or glutamate receptors between T- and S-neurons, except for *Gabrr2* and *Grik1,* which were lower and higher in the S-neurons, respectively (S3 Table). Third, *Adrb1* (adrenergic receptor, β-1) was expressed at higher levels in S-neurons than in T-neurons (Fig 3D and S3 Table). Fourth, T- and S-neurons presented distinct cell-adhesion molecule (CAM) expression profiles, suggesting different cell-surface assembly codes (Fig 3E and S3 Table). Taken together, the above RNA expression profile suggests that T-neurons and S-neurons are two distinct neuronal populations.

The above differences in the mRNA profiles of T- and S-neurons predict differences in their electrophysiological properties. We thus made patch-clamp recordings from identified T- and S-neurons in the dmPFC slices and compared their electrophysiological properties. We observed significantly greater input resistance in the S-neurons (Fig 3F). Excitatory inputs, measured using sEPSCs, were not different between T- and S-neurons (Figs 3G and S3). Interestingly, T-neurons presented a conditioning-dependent increase in sEPSC frequency (Fig 3G), which likely reflects an increased probability of presynaptic release since the sEPSC amplitude was unaltered (S3A Fig). The intrinsic excitability was not different between T- and S-neurons (Fig 3H). The half-width of spikes was significantly greater in the S-neurons (Fig 3I), and spike amplitudes were significantly lower in the S-neurons (Figs 3J and S3F), whereas most other properties of spikes were not different (S3B–S3E Fig). The spike amplitude in the S-neurons also showed a conditioning-dependent increase.

### Sustained S-neuron responses and freezing require TeA inputs and dmPFC NE elevation

**Fig 4:**
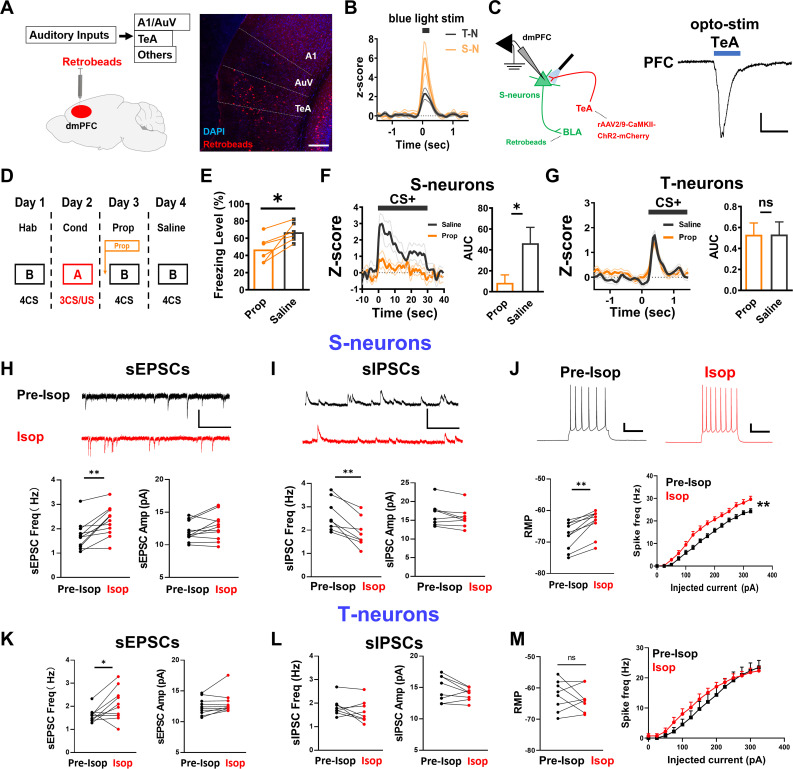
Contribution ofβ-norepinephrine receptors to dmPFC neuronal responses and freezing levels. **(A)** Experimental paradigm for examining inputs to dmPFC using injection of retrobeads in dmPFC (left) and visualization of retrobeads in the auditory cortex (right). A1, primary auditory cortex; AuV, auditory cortex; TeA, temporal association cortex. Scale bar, 200 μm. **(B)** Responses (*z*-score) of T-neurons and S-neurons to opto-activation of TeA inputs (0.9 Hz, 250 ms). T-neurons, *n* = 20 units/5 mice; S-neurons, *n* = 12 units/5 mice. rAAV2/9-CaMKII-ChR2-mCherry viruses were injected in TeA and optical fiber implanted in the TeA to activate TeA neurons. **(C)** Injection of rAAV2/9-CaMKII-ChR2-mCherry virus in the TeA (Left). Representative EPSC trace in the dmPFC neurons to opto-stimulation of TeA inputs (Right). Bar indicates blue light illumination. Scale bars, 200 pA and 25 ms. **(D)** Experimental procedure for examining intra-cerebroventricular injection of propranolol or saline on neuronal responses and freezing levels. **(E)** Freezing levels during retrieval with (Prop) or without (Saline) propranolol injection in conditioned mice. Two-tailed paired *t* test, Prop vs. Saline, *P* < 0.05; *N* = 6 mice. **(F)** Effect of intra-cerebroventricular injection of Prop on spike rates in the S-neurons. Two-tailed paired *t t*est, Prop vs. Saline, *P* < 0.05. *N* = 9 units/6 mice. **(G)** Effect of intra-cerebroventricular injection of Prop on spike rates in the T-neurons. *N* = 22 units/6 mice. **(H)** (Top) Representative sEPSCs traces in the S-neurons during bath application of Isop (50 μM). Scale bars, 20 pA and 50 ms. (Bottom) Effects of Isop on sEPSC frequency (Two-tailed paired *t* tes*t*, *t* = 4.246, *df* = 10, Pre-Isop vs. Isop, *P* < 0.01; *n* = 11 cells/4 mice), and sEPSC amplitude in the S-neurons (Two-tailed paired *t* test, *t* = 0.976, *df* = 20, Pre-Isop vs. Isop, *P* > 0.05; *n* = 11 cells/4 mice). **(I)** (Top) Representative sIPSCs traces in S-neurons during bath application of Isop. Scale bars, 50 pA and 50 ms. (Bottom) Effects of Isop on sIPSC frequency (Two-tailed paired *t* test, *t* = 4.961, *df* = 7, Pre-Isop vs. Isop, *P* < 0.01; *n* = 8 cells/3 mice) and sIPSC ampli*t*ude in the S-neurons (Two-tailed paired *t* test, *t* = 2.088, *df* = 7, Pre-Isop vs. Isop, *P* > 0.05; *n* = 8 cells/3 mice). **(J)** (Top) Representa*t*ive action potential traces in the S-neurons before and after bath application of Isop. Scale bars, 20 mV and 200 ms. (Lower left) Effects of Isop on the resting membrane potential (RMP) in the S-neurons (Two-tailed paired *t* test, *t* = 6.143, *df* = 9, Pre-Isop vs. Isop, *P* < 0.01; *n* = 10 cells/4 mice) and intrinsic excitabili*t*y of S-neurons (Two-way RM ANOVA, *F* (15, 288) = 2.294, Bonferroni’s posttest, *P* < 0.01; Pre-Isop vs. Isop, *P* < 0.01; *n* = 12 cells/3 mice). **(K)** Effects of Isop on sEPSC frequency (Two-tailed paired *t* test, *t* = 2.333, *df* = 9, Pre-Isop vs. Isop, *P* < 0.05; *n* = 10 cells/4 mice) and sEPSC amplitude in the T-neurons (Two-tailed paired *t* tes*t*, *t* = 1.758, *df* = 9, Pre-Isop vs. Isop, *P* < 0.05; *n* = 10 cells/4 mice). **(L)** Effects of Isop on sIPSC frequency (Two-tailed paired *t* test, *t* = 1.265, *df* = 7, Pre-Isop vs. Isop, *P* > 0.05; *n* = 8 cells/3 mice) and sIPSC ampli*t*ude in the T-neurons (Two-tailed paired *t* test, *t* = 1.778, *df* = 7, Pre-Isop vs. Isop, *P* > 0.05; *n* = 8 cells/3 mice). **(M)** Effec*t*s of Isop on RMP (Two-tailed paired *t* test, Pre-Isop vs. Isop, *P* > 0.05; *n* = 8 cells/3 mice) and intrinsic excitability of T-neurons (Two-way RM ANOVA, *F* (15, 336) = 0.4195, Bonferroni’s pos*t*test, *P* > 0.05; Pre-Isop vs. Isop, *P* > 0.05; *n* = 12 cells/3 mice). Numerical data can be found in S1 Data.

The observations that S-neuron responses match well with the CS durations (Fig 1E) and that opto-activation of axonal terminals onto T-neurons resulted in only a transient response in the S-neurons (Fig 2I) suggest that sensory (sound) inputs contribute to the CS-elicited sustained responses in the S-neurons. Previous studies have shown that the temporal association cortex (TeA) mediates the specific type of auditory conditioning stimuli used in our experiments [47–49]. To examine whether TeA represents the primary auditory input to the dmPFC, we first injected retrobeads into the dmPFC and observed that the majority of the beads were present in TeA but not in the primary auditory cortex (Fig 4A). Optogenetic stimulation of TeA inputs resulted in strong spiking in the dmPFC S-neurons and smaller responses in the T-neurons *in vivo* (Fig 4B). In addition, opto-stimulation of TeA inputs in PFC slices elicited strong responses in the identified S-neurons (on the basis of their projection to the BLA, green, Retro^+^ neurons) (Fig 4C). Both observations support strong TeA inputs onto the dmPFC S-neurons.

We next investigated the mechanism underlying the transition from transient responses to sustained responses in dmPFC neurons, or in other words, the mechanism required to sustain the responses in S-neurons to match the CS+ duration. Previous studies have shown that NE signaling via β-adrenergic receptors maintains S-neuron responses and freezing [16,17]. The intracerebroventricular injection of the β-noradrenergic antagonist propranolol (Prop, Fig 4D) resulted in a significant reduction in both freezing levels (Fig 4E) and S-neuron spiking (Fig 4F) elicited by CS+. Importantly, T-neuron spiking was not significantly affected by Prop (Fig 4G), indicating that NE modulation is specific to S-neurons and also highlighting the necessity for S-neuron responses in the generation of freezing behavior.

NE is known to increase the activity/excitability of postsynaptic neurons and neurotransmitter release [50–53]. Our RNA-seq results also suggested increased expression of β-adrenergic receptors in S-neurons (Fig 3D). Therefore, we tested whether NE, especially the activation of β-ARs, preferentially modulates the properties of S-neurons. To do so, we bath applied the β-AR agonist isoprenaline (ISOP, 50 μM), which led to a significant increase in sEPSC frequency but not amplitude (Fig 4H) in the identified S-neurons in the PFC slices, suggesting increased presynaptic glutamate release. ISOP also induced a significant reduction in sIPSC frequency but not amplitude (Fig 4I), significant membrane depolarization and a significant increase in intrinsic excitability (Fig 4J), in S-neurons. Collectively, these results indicate that NE enhances S-neuron activity via increased excitatory inputs, decreased inhibitory inputs, and increased intrinsic neuronal excitability. Thus, CS-elicited NE release in the dmPFC is likely to promote greater responsivity in S-neurons during threat memory retrieval. In contrast, the main impact of ISOP on T-neurons was increased sEPSC frequency (Figs 4K–4M and S4). Collectively, these results indicate a preferential enhancement of S-neuron activity by NE via increased excitatory inputs, decreased inhibitory inputs, and increased intrinsic neuronal excitability. Thus, CS-elicited NE release in the dmPFC is likely to promote greater responsivity in S-neurons during threat memory retrieval.

### Contribution of dmPFC NE elevation to freezing behavior

**Fig 5:**
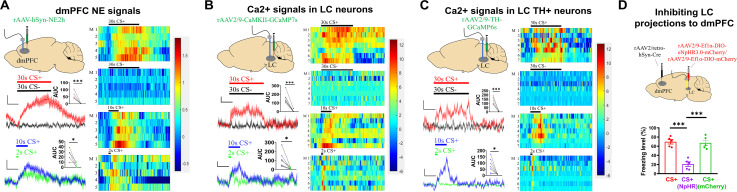
The dmPFC NE levels, LC neuron activity and BLA NE levels associated with CS retrieval. **(A)** (Left) Sites of virus injections and NE signal recording (top), and plots of NE signals with stimuli (middle and bottom). Scale bars, 0.5% *ΔF*/*F* and 10 s. Inserts: AUC of *ΔF*/*F* of dmPFC NE signals. Two-tailed unpaired *t* test, 30 s CS+ vs. 30 s CS−, *P* < 0.001; 10 s CS+ vs. 2 s CS+, *P* < 0.05. (Right) Heat maps showing dmPFC NE signals elicited by 30 s CS+, 30 s CS−, 10 s CS+ and 2 s CS+. *N* = 5 mice per group. **(B)** (Left) Sites of virus injections and Ca^2+^ signal recording (top), and plots of Ca^2+^ signals with stimuli (middle and bottom). Scale bars, 2% *ΔF*/*F* and 10 s. Inserts: AUC of *ΔF*/*F* of LC Ca^2+^ signals. Two-tailed unpaired *t* tes*t*, 30 s CS+ vs. 30 s CS−, *P* < 0.01; 10 s CS+ vs. 2 s CS+, *P* < 0.05. (Right) Heat maps of Ca^2+^ responses in the LC-neurons in response to 30 s CS+, 30 s CS−, 10 s CS+ and 2 s CS+. *N* = 7 mice per group. **(C)** (Left) Sites of virus injections and optical fiber recording (top), and plots of Ca^2+^ signals in the LC TH^+^ neurons with stimuli (middle and bottom). Scale bars, 2% *ΔF*/*F* and 10 s. Insert: AUC of *ΔF*/*F* of Ca^2+^ signals in the LC TH^+^ neurons. Two-tailed unpaired *t* test, 30 s CS+ vs. 30 s CS−, *P* < 0.01; 10 s CS+ vs. 2 s CS+, *P* < 0.05. (Right) Hea*t* maps for Ca^2+^ signals in the LC TH^+^ neurons in response to 30 s CS+, 30 s CS−, 10 s CS+ and 2 s CS+. *N* = 7 mice per group. **(D)** Sites of virus injections and optical fiber recording (left), and impact of opto-inhibiting dmPFC-projecting LC neurons on freezing levels (right). Two-tailed paired *t* test, CS+ vs. CS+ (NpHR), *P* < 0.001; CS+ (NpHR) vs. CS+ (mCherry), *P* < 0.001. *N* = 5 mice, each group. Numerical data can be found in S1 Data.

Our results thus far suggest that NE plays an important role in the transition from memory to behavior. Previous studies have indicated that conditioned threat cues increase dmPFC NE levels [54]. To directly monitor changes in dmPFC NE levels, we injected rAAV-hSyn-NE2h virus (NE fluorescence sensor) [55–57] into the dmPFC and monitored extracellular NE levels using fiber photometry (Fig 5A). In the conditioned mice, 30 s or 10 s CS+ led to a rapid and significant increase in NE sensor fluorescence, which decayed slowly toward the pre-CS level after CS termination (Fig 5A). In contrast, a 30 s CS− (which did not elicit significant freezing) did not elicit a significant change in fluorescence in the same mice (Fig 5A), indicating that PFC NE elevation is specific to the CS+. In comparison, a 2 s CS+ (mimicking T-neuron activation; Fig 1B) or 10 s CS+ led to a shorter increase in NE sensor fluorescence, with a duration elicited by a 2 s CS+ longer than the CS+ duration (Fig 5A). We observed similar response dynamics using a sensor with medium NE affinity (rAAV-hSyn-NE2m) [57] (S5 Fig), indicating that the affinity of the NE sensor does not significantly affect the dynamics of the observed NE sensor fluorescence. Although our NE fluorescence signals cannot be readily converted into NE concentrations, the extracellular NE concentration has been shown to be linearly related to the LC output across a range of tonic LC activities [58,59].

A major source of dmPFC NE comes from the LC [60–63]. In support of this, we observed a significant increase in Ca^2+^ signals at 30 and 10 s CS+ but not at 30 s CS− in the LC neurons expressing the rAAV2/9-CaMKII-GCaMP7s virus (Fig 5B) [58]. In addition, significant increases in the Ca^2+^ signals were elicited by 2 s CS+ or 10 s CS+ (Fig 5B). Since the majority of NE-releasing LC neurons express tyrosine hydroxylase (TH) [64], we monitored Ca^2+^ activity in the LC TH^+^ neurons using injection of rAAV2/9-TH-GCaMP6s virus in the LC and observed similar response patterns (Fig 5C). These results indicate that CS+ elicits a strong, selective NE elevation in the dmPFC and that this elevation roughly matches the CS+ duration. This dmPFC NE elevation is likely mediated by the activation of dmPFC-projecting LC neurons. To determine whether dmPFC NE elevation is required for freezing behavior, we inhibited LC-to-dmPFC projections by injecting rAAV2/retro-hSyn-Cre virus into the dmPFC and rAAV2/9-Ef1α-DIO-eNpHR3.0-mCherry virus or rAAV2/9-Ef1α-DIO-mCherry virus (control virus) into the LC (Fig 5D). We found that yellow light illumination resulted in freezing levels that were significantly lower than those elicited by the CS+ or freezing methods in mice expressing the control virus and subjected to the same light illumination (Fig 5D). This effect was similar to that induced by Prop injection (Fig 4E), supporting the critical contribution of LC to dmPFC projections to freezing behavior. LC neurons that project to the BLA are also activated by CS+ after threat-conditioning [23,61], and we observed strongly elevated NE signals in the BLA in response to 30 s CS+ but not CS− (S6 Fig).

### LC–NE neurons receive inputs from the dmPFC and TeA

**Fig 6:**
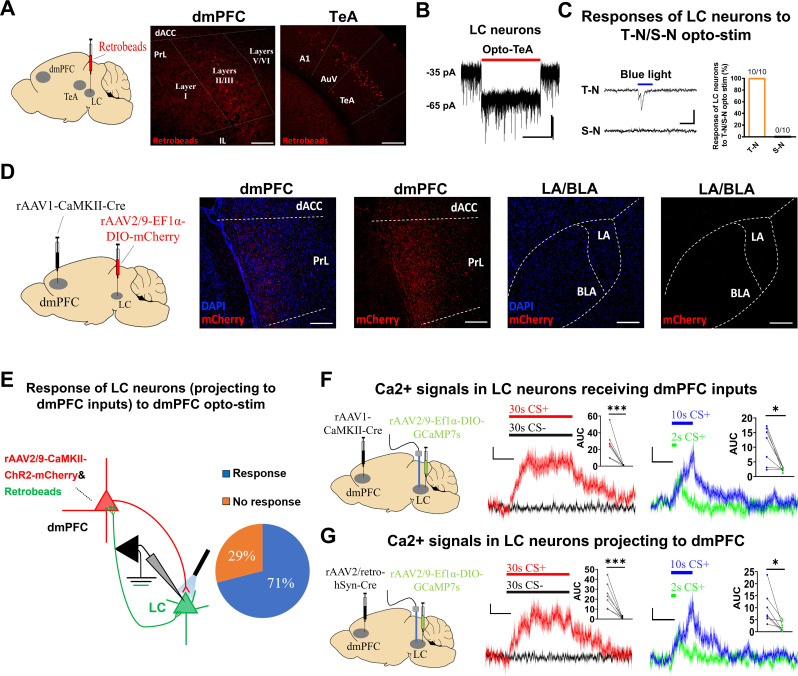
LC/NE neurons are activated by inputs from dmPFC and TeA, and project to dmPFC. **(A)** (Left) Experimental paradigm for examining major brain regions that project to LC, using retrobeads (red) injected in the LC. (Right) Brain regions with red fluorescence, dmPFC and TeA. Scale bar, 200 μm. **(B)** Responses of the LC neurons in LC slice to opto-activation of axonal terminals from TeA neurons (red bar, 10 s). rAAV2/9-CaMKII-ChR2-mCherry virus injected in the TeA, and laser illumination in the slices. Scale bars, 20 pA and 5 s. **(C)** (Left) Representative responses of LC neurons to opto-activation (bar, 50 ms) of axonal terminals from either T-neurons or S-neurons. Scale bars, 20 pA and 50 ms. (Right) All LC neurons responded to opto-activation of axonal terminals from the T-neurons, but none responded to opto-activation of axonal terminals from the S-neurons. *n* = 10 cells. **(D)** (Left) Experimental paradigm for examining outputs from the LC neurons receiving dmPFC projections. (Right) Presence of the mCherry fluorescence (red) in the dmPFC but not LA or BLA. Scale bars, 200 μm. **(E)** (Left) Schematic diagram showing opto-activation of dmPFC neurons axon terminals and recording in the dmPFC-projecting LC neurons in LC slices. (Right) 71% of dmPFC-projecting LC neurons responded to opto-activation of dmPFC neuron axon terminals and 29% showed no response to the same stimuli. **(F)** (Left) Diagram for virus injections, (middle and right) Ca^2+^ responses elicited by 30 s CS+, 30 s CS−, 10 s CS+ and 2 s CS+ in the LC neurons receiving dmPFC inputs. Scale bars, 0.5% *ΔF*/*F* and 10 s. *N* = 7 mice. Insert: AUC of *ΔF*/*F* of Ca^2+^ signals. Two-tailed unpaired *t* test, 30 s CS+ vs. 30 s CS−, *P* < 0.001; 10 s CS+ vs. 2 s CS+, *P* < 0.05. **(G)** (Left) Diagram for virus injections, (middle and right) Ca^2+^ responses to 30 s CS+, 30 s CS−, 10 s CS+ and 2 s CS+ in the LC neurons projecting to dmPFC. Scale bars, 0.5% *ΔF*/*F* and 10 s. *N* = 7 mice. Insert: AUC of *ΔF*/*F* of Ca^2+^ signals. Two-tailed unpaired *t* tes*t*, 30 s CS+ vs. 30 s CS−, *P* < 0.001; 10 s CS+ vs. 2 s CS+, *P* < 0.05.

As dmPFC neurons are activated during CS+ presentation, this activation may in turn activate LC-neurons [65–68]. In addition, the time-locked responses of the LC-neurons suggest that they may be activated by sound inputs. In support of this idea, we observed prominent fluorescence in the dmPFC and TeA but not in the LA or BLA in the mice injected with retrobeads (red) in the LC (Figs 6A and S7). By expressing the rAAV2/9-CaMKII-ChR2-mCherry virus in TeA, we observed robust responses in LC neurons to opto-activation of TeA axonal terminals in the LC slices (Fig 6B). Since both T-neurons and S-neurons are present in the dmPFC, we asked whether their inputs to LC neurons may differ. To this end, we labeled the dmPFC neurons receiving LA inputs with mCherry (mCherry^+^, rAAV1-CaMKII-Cre virus injected in the LA and rAAV2/9-Ef1α-DIO-mCherry virus in the dmPFC) and LC-projecting dmPFC neurons with CTB 488 (CTB 488^+^, injected in the LC). Most of the mCherry^+^ and CTB 488^+^ neurons overlapped in the dmPFC, and the ratio of mCherry^+^ neurons to CTB 488^+^ neurons was 0.835. This observation confirms an overlapping population within the dmPFC that both receives LA inputs and projects to the LC (S8 Fig).

To confirm this finding using electrophysiological recording, we recorded LC neuron responses to selective opto-stimulation of inputs from either T-neurons (their receiving LA inputs; rAAV1-CaMKII-Cre virus was injected into the LA, and rAAV2/9-Ef1α-DIO-ChR2-mCherry virus was injected into the dmPFC to label T-neurons) or S-neurons (their projections to the BLA; rAAV2/retro-hSyn-Cre virus was injected into the BLA, and the rAAV2/9-Ef1α-DIO-ChR2-mCherry virus was injected into the dmPFC to label S-neurons). Strong light-evoked EPSCs in the LC neurons stimulated T-neuron inputs (10/10), but no responses were observed with the stimulation of S-neuron inputs (0/10) (Fig 6C). This result indicates that the dmPFC T-neuron inputs constitute the major PFC inputs to LC neurons in the context of threat memory.

As the dmPFC projects to the LC and the LC projects back to the dmPFC, we examined whether the same LC-neurons receive dmPFC inputs and project back to the dmPFC. For this analysis, mice were injected with the rAAV1-CaMKII-Cre virus in the dmPFC and the rAAV2/9-Ef1α-DIO-mCherry virus in the LC, and the presence of fluorescently labeled axon terminals indicated projections from the LC to the dmPFC (Fig 6D). In contrast, no fluorescence was observed in the LA or BLA, indicating that no strong projections to the LA or BLA from LC neurons received dmPFC inputs (Fig 6D). To further confirm these observations via electrophysiological recordings, we examined the responses of dmPFC-projecting LC neurons to the opto-stimulation of dmPFC inputs (axonal terminals) in LC slices. For this analysis, the rAAV2/9-Ef1α-CaMKII-ChR2-mCherry virus and retrobeads (green) were injected into the dmPFC. Approximately 71% of dmPFC-projecting LC neurons showed strong responses to the opto-activation of dmPFC neuron axonal terminals, and 29% showed no responses (Figs 6E and S9). Given that some connections are likely severed during the slicing procedure, this result indicates that the majority of LC neurons are reciprocally connected to dmPFC neurons.

We next examined the Ca^2+^ responses of the LC neurons receiving dmPFC inputs and observed robust responses to 2, 10, and 30 s CS+ but not to 30 s CS− (Figs 6F and S10). We also observed strong CS+-elicited Ca^2+^ responses in the LC neurons that project to dmPFC inputs *in vivo*, which roughly matched the CS duration (Figs 6G and S10). In addition, inhibiting dmPFC-to-BLA projections significantly reduced freezing levels without affecting dmPFC NE signals, whereas freezing levels in mice expressing control virus were not affected by CNO (S11 Fig), which is consistent with these projections being downstream of dmPFC-NE signaling.

### An increase in the dmPFC NE level opens a short window for the transition to behavior during memory retrieval

Our results thus far have demonstrated that dmPFC NE levels are significantly elevated during CS+-triggered threat memory retrieval, and this elevation enhances the activity in the dmPFC S-neurons to enable their sustained spiking during CS+.

In addition, increased LC–NE neuronal activity and the resulting increased dmPFC NE levels are required for sustained S-neuron responses and freezing. We therefore next asked whether higher dmPFC NE levels sufficient to drive these responses and behaviors.

**Fig 7:**
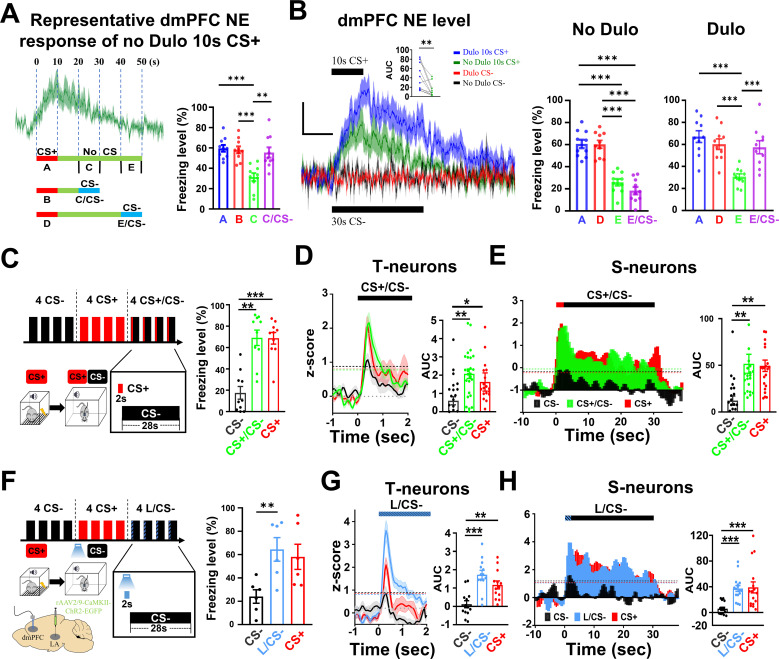
An increase in dmPFC NE level opens a short window for transition to behavior during memory retrieval. **(A)** (Left, top) Representative dmPFC NE responses to 10 s CS+ in the absence of Dulo. (Left bottom) Diagram for testing the impact of CS− on freezing levels when they were given at different time points after termination of 10 s CS+. Blue regions mark the period when CS− was given. (Right) Freezing levels during the corresponding time periods. Two-tailed paired *t* test, A vs. C, *P* < 0.001; B vs. C, *P* < 0.001; C vs. C/ CS−, *P* < 0.01. The same set of mice was used, *N* = 10 mice. **(B)** (Left) dmPFC NE signals elicited by 10 s CS+ and 30 s CS− in the absence or presence of NE uptake inhibitor Dulo. Insert: AUC of *ΔF*/*F* of dmPFC NE signals. Two-tailed unpaired *t* test, Dulo vs. No Dulo, *P* < 0.01. (Middle) Freezing levels wi*t*hout Dulo injection. Two-tailed paired *t* test, A vs. E, *P* < 0.001; A vs. E/CS−, *P* < 0.001; D vs. E, *P* < 0.001; D vs. E/CS−, *P* < 0.001. (Right) Freezing levels wi*t*h Dulo injection. Two-tailed paired *t* test, A vs. E, *P* < 0.001; D vs. E, *P* < 0.001; E vs. E/CS−, *P* < 0.001. Scale bars, 0.5% *ΔF*/*F* and 10 s. The same set of mice was used, *N* = 10 mice. **(C)** Experimental protocol and stimulus pa*tt*erns used (left) and corresponding freezing levels (right). One-way RM ANOVA, *F* (2, 8) = 0.163, Bonferroni’s posttest; CS− vs. CS+/CS−, *P* < 0.01; CS− vs. CS+, *P* < 0.001; *N* = 10 mice. **(D)** Spike rates in the T-neurons during CS retrieval (left) and AUC (*z*-score, 2 s) (right). One-way RM ANOVA, *F* (2, 72) = 4.699, Bonferroni’s posttest; CS− vs. CS+/CS−, *P* < 0.01; CS− vs. CS+, *P* < 0.05; *n* = 25 units/10 mice. **(E)** Spike rates in the S-neurons during CS retrieval (left) and AUC (*z*-score, 30 s) (right). One-way RM ANOVA, *F* (2, 33) = 2.092, Bonferroni’s posttest; CS− vs. CS+/CS−, *P* < 0.01; CS− vs. CS+, *P* < 0.01; *n* = 12 units/10 mice. **(F)** Experimental protocol for CS retrieval test, by CS+, CS−, by a hybrid stimulus (L/CS−) with opto-activation of LA axonal terminals in dmPFC for 2 s followed immediately by CS− (left) and corresponding freezing levels during retrieval (right). One-way RM ANOVA, *F* (2, 18) = 3.807, Bonferroni’s posttest; CS− vs. L/CS−, *P* < 0.05; *N* = 6 mice. **(G)** Spike rates in the T-neurons (left) and AUC (*z*-score, 2 s) (right). One-way RM ANOVA, *F* (2, 42) = 6.67, Bonferroni’s posttest; CS− vs. L/CS−, *P* < 0.001; CS− vs. CS+, *P* < 0.05; *n* = 13 units/6 mice. **(H)** Spike rates in the T-neurons (left) and AUC (*z*-score, 30 sec) (right). One-way RM ANOVA, *F* (2, 18) = 5.099, Bonferroni’s posttest; CS− vs. L/CS−, *P* < 0.001; CS− vs. CS+, *P* < 0.001; *n* = 16 units/6 mice. Numerical data can be found in S1 Data.

The NE fluorescence signals elicited by 10 s CS+ took approximately 30 s to decay to the baseline level (Fig 7A), and the duration of freezing was approximately 10 s (Fig 1G). This observation suggests that CS+ but not NE levels control whether freezing occurs. To test this hypothesis, we presented a 10 s CS− starting either 10 s or 30 s after CS+ termination (sections C or E, respectively; Fig 7A). The rationale for using CS− was that it does not elicit significant freezing or NE elevation by itself, but S-neurons are expected to respond to CS− since they show vigorous responses to the activation of TeA neurons (Fig 4B). Interestingly, CS− presented significant freezing at 10 s but not at 30 s after CS+ termination (Fig 7A and 7B). This finding suggests that the dmPFC NE levels at 10 s after CS+ termination (or 20 s from the start of the CS+, section C in Fig 7A) can enhance S-neuron responses only if a CS is present. In contrast, the NE level at 30 s after CS+ termination (section E, Fig 7B) was not sufficient. If a certain dmPFC NE level can elicit freezing with the presence of a CS, we hypothesized that it may be possible to alter the duration of the freezing response window by manipulating the NE levels. The extracellular NE levels are modulated by NE transporters, and injection of the NE uptake blocker duloxetine (Dulo) resulted in higher NE levels and slower decay to baseline (Fig 7B). This enhancement by Dulo was limited to the CS+ as the CS− did not affect NE levels (Fig 7B), likely because the CS− does not induce significant activation of LC neurons (Fig 5B and 5C). We then tested whether significant freezing could be elicited by the CS− at 30 s after CS+ termination in the presence of Dulo (section E, Fig 7A) and significant freezing was observed (Fig 7B).

The above observations indicate that to sustain S-neuron responses and enable freezing behavior, both TeA (CS) inputs and sufficiently high dmPFC NE levels are needed. These observations also suggest that transient activation of dmPFC neurons may be sufficient to elicit significant elevation of dmPFC NE levels, which can be sustained by an ensuing CS (including the CS−). If this is true, a brief CS+ followed by a CS− should be sufficient to elicit significant freezing levels. To test this possibility, we used a hybrid CS signal: a brief (2 s) CS+ signal followed immediately by a CS− signal (28 s) (Fig 7C). This hybrid CS elicited freezing levels similar to those elicited by 30 s CS+ (Fig 7C) and spiking in both T-neurons (Fig 7D) and S-neurons (Fig 7E), with similar amplitudes and durations to those elicited by 30 s CS+ in the same set of mice. To exclude the possibility that the non-dmPFC neurons activated by the 2 s CS+ may have a large contribution to the above effects of the hybrid CS, we used opto-activation of LA axonal terminals in the dmPFC. Replacing the 2 s CS+ with a 2 s opto-activation of LA axonal terminals in the dmPFC (L/CS− hybrid, Fig 7F) resulted in freezing levels (Fig 7F) and spiking in T-neurons (Fig 7G) and S-neurons (Fig 7H) that were indistinguishable from those elicited by the CS+ in the same set of mice. Notably the frequency and pattern used for the CS− in these experiments were very different from those used for the CS+ experiments, and the CS− alone did not elicit significant freezing. Control experiments using light or 2 s CS+ alone were not conducted since 2 s is too short to measure freezing levels accurately. Taken together, these findings strongly indicate that brief CS+ presentation initiates dmPFC neuron responses and freezing responses to nonconditioned CS.

### Short-term plasticity of the dmPFC-LC connections enables CS generalization

**Fig 8:**
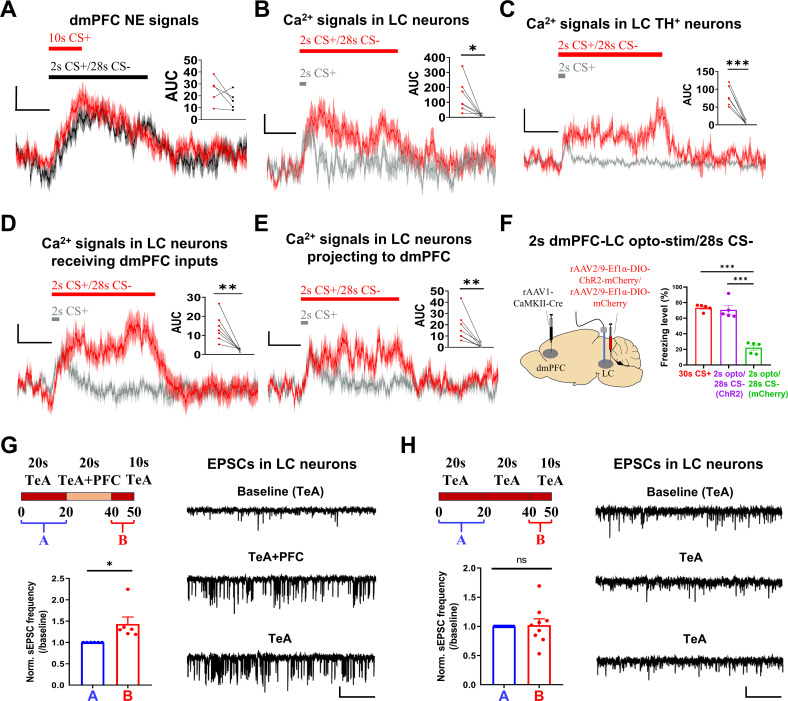
Short-term plasticity of the dmPFC-LC connections enables the CS generalization during threat memory retrieval. **(A)** NE signals in the dmPFC elicited by 10 s CS+ or 2 s CS+/28 s CS−. Scale bars, 0.5% *ΔF*/*F* and 10 s. *N* = 5 mice. Insert: AUC of *ΔF*/*F* of dmPFC NE signals. **(B)** LC Ca^2+^ signals elicited by 2 s CS+ or 2 s CS+/28 s CS−. Scale bars, 2% *ΔF*/*F* and 10 s. *N* = 7 mice. Insert: AUC of *ΔF*/*F* of Ca^2+^ signals. Two-tailed unpaired *t* test, 2 s CS+/28 s CS− vs. 2 s CS+, *P* < 0.05. **(C)** Ca^2+^ signals in the LC TH^+^ neurons elicited by 2 s CS+ and 2 s CS+/28 s CS−. Scale bars, 2% *ΔF*/*F* and 10 s. *N* = 7 mice. Insert: AUC of *ΔF*/*F* of Ca^2+^ signals. Two-tailed unpaired *t t*est, 2 s CS+/28 s CS− vs. 2 s CS+, *P* < 0.001. **(D)** Ca^2+^ signals elicited by 2 s CS+/28 s CS− and 2 s CS+ in the LC neurons receiving dmPFC projections. *N* = 7 mice, Two-tailed unpaired *t* tes*t*, 2 s CS+/28 s CS− vs. 2 s CS+, *P* < 0.01. **(E)** Ca^2+^ signals elicited by 2 s CS+/28 s CS− and 2 s CS+ in the LC neurons projecting to dmPFC. *N* = 7 mice, Two-tailed unpaired *t* test, 2 s CS+/28 s CS− vs. 2 s CS+, *P* < 0.01. **(F)** (Lef*t*) Sites of virus injections and optical fiber implantation. (Right) Freezing levels elicited by 30 s CS+, 2 s blue light stimulation followed by 28 s CS−. *N* = 5 mice, each group. **(G)** (Left) (Top) Experimental procedure for opto-stimulation. (Bottom) Normalized sEPSC frequency in the LC neurons to the sEPSCs during baseline period (period **A)**. (Right) Representative traces of recorded EPSCs in the LC neurons during baseline (TeA stimulation only), during TeA + PFC stim and after stimulation (TeA stimulation only). Scale bars, 2 s and 20 pA. *N* = 6 cells/2 mice. **(H)** (Left) (Top) Experimental procedure for opto-stimulation. (Bottom) Normalized sEPSC frequency in the LC neurons to the sEPSC during baseline period (period **A)**. (Right) Representative traces of recorded EPSCs in the LC neurons to opto-stimulation of TeA inputs, for the same durations as in **(G)**. Scale bars, 2 s and 20 pA. *N* = 9 cells/2 mice. Numerical data can be found in S1 Data.

We next investigated the mechanism underlying the CS generalization we observed with hybrid CS. Since dmPFC NE levels play a critical role in the above processes, we first asked whether LC neuron activity is high and sustained during the 2 s CS+/28 s CS−. Indeed, dmPFC NE fluorescence signals elicited by the 2 s CS+/28 s CS− remained high throughout the entire CS presentation (Figs 8A and S12). The dmPFC NE signals were similar between 2 s CS+/28 s CS− and 10 s CS+, but the freezing levels were much longer for the former, which is consistent with the presence of CS− in the former case, and the CS is required to sustain S-neuron responses. We also observed high and sustained LC-neuron responses to 2 s CS+/28 s CS− (Figs 8B and S12). Consistently, sustained Ca^2+^ signals in the TH^+^-LC neurons were also observed during 2 s CS+/28 s CS− (Figs 8C and S12) and in the LC neurons receiving dmPFC inputs (Figs 8D and S10) or projecting to the dmPFC (Figs 8E and S10). A comparison of the Ca^2+^ responses to 2 s CS+ and hybrid CS suggested that the initial responses were similar between these two CSs and that the sustained phase of hybrid CS likely sustains S-neuron responses (Fig 8B–8E). Thus, we mimicked this initial 2 s CS+ activation using a 2 s opto-activation of LC neurons that receive dmPFC inputs followed by 28 s of CS− and observed freezing levels similar to those following 30 s CS+ but no elevated freezing levels in mice expressing the control virus (Fig 8F). This finding suggests a critical role, likely gating in nature, of dmPFC inputs in enabling high and sustained responses to CS− in LC neurons.

Since LC neurons show experience-driven plasticity [21], we tested whether brief dmPFC inputs may enhance the responses of LC neurons to TeA (sound) inputs. For this analysis, we recorded sEPSCs in LC neurons that respond to the opto-activation of both dmPFC and TeA inputs. After establishing a stable baseline response to TeA opto-stimulation, we administered either 20 s of combined dmPFC + TeA stimulation (Fig 8G) or TeA stimulation alone as a control (Fig 8H). Only in the case of stimulating both dmPFC and TeA inputs were significantly greater responses to TeA inputs observed, and this increase lasted for at least 10 s in the absence of dmPFC stimulation. Thus, the short-term plasticity of the dmPFC-LC system may enable CS generalization by enhancing LC neuron responses to sounds/CSs (including nonconditioned CSs).

## Discussion

**Fig 9:**
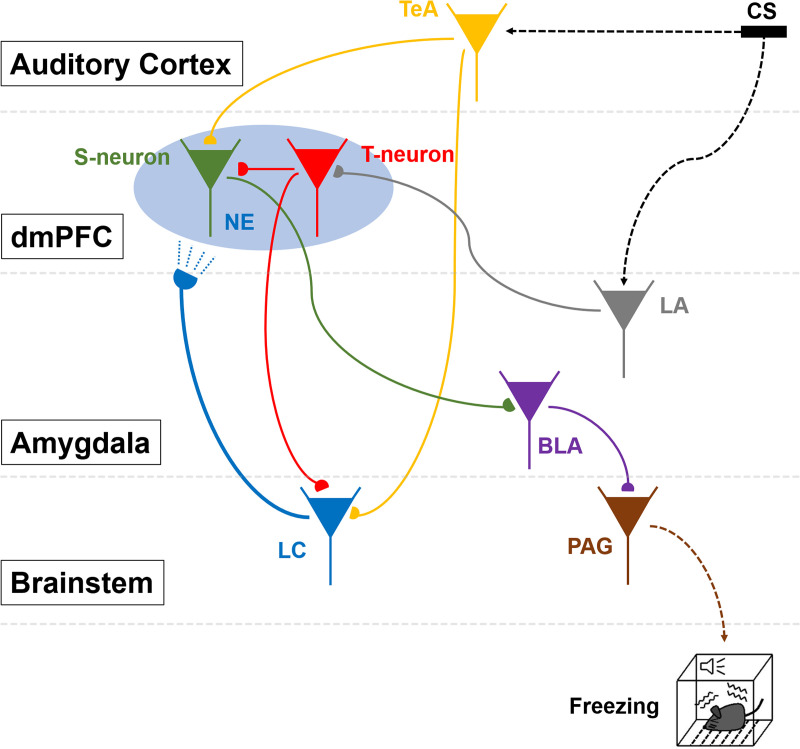
Summary on the neural circuit mediating the transition from memory retrieval to freezing behavior. Presentation of CS+ activates LA neurons and TeA neurons. The activated LA neurons activate dmPFC T-neurons, which in turn activate dmPFC S-neurons and LC neurons. S-neurons receive inputs from TeA and send their outputs to BLA, which projects to PAG to enable freezing behavior. Activated LC neurons release NE in the dmPFC via their projections.

In this study, via a combination of methods and a simple, robust and widely used threat memory model system, we addressed a general question of how memory elicits behavior. We demonstrated that the sustained responses of dmPFC S-neurons are required to initiate freezing behavior, the duration of which matches that of the CS. The dmPFC T-neurons and TeA auditory inputs, both of which are activated by the CS, increase activity in the LC–NE neurons, and these activated LC–NE neurons project to the dmPFC and release NE. Elevated dmPFC NE levels enable sustained responses in S-neurons. These highly interactive processes ensure that behavioral responses are specific to the conditioned/learned cues but can also be modulated to fit the emotional and physical states of an organism during memory retrieval for optimal adaptation. A summary is shown in Fig 9.

At the beginning of the study, we defined T-neurons and S-neurons as dmPFC excitatory neurons that exhibit transient and sustained responses to threat CS, respectively. Our current findings confirm that T- and S-neurons are two distinct dmPFC populations that differ in the following ways: (1) Inputs: S-neurons receive inputs from the TeA and T-neurons, whereas T-neurons receive inputs from the LA and local GABAergic neurons [14,15]. (2) Outputs: S-neurons project to the BLA, whereas T-neurons project to S-neurons and the LC. (3) Electrophysiological properties: S-neurons have greater input resistance, longer spike half-widths, and lower spike amplitudes. (4) Genes: T- and S-neurons show differential expression levels of Ca^2+^ channels, cell-adhesion molecules and β-ARs. (5) Localization: T- and S-neurons occupy nonoverlapping locations in the dmPFC but are intermingled with each other. The above differences align with their distinct contributions to the transition from memory to behavior.

It is well established that the LC–NE system is important for the formation, consolidation, and extinction of conditioned threat responses [21–23,69]. Here, we have extended the role of the LC–NE system to include the transition from memory to behavior generation. After confirming that elevated dmPFC NE levels are required for dmPFC S-neuron responses and freezing behavior, we showed that LC–NE neurons are activated by dmPFC inputs during CS+. These activated LC neurons in turn project back to the dmPFC [66,67,70–72]. The resulting high dmPFC NE levels enhance the activity of S-neurons via β-ARs, which is consistent with findings in mice [16,17] and humans [73] and the expression pattern of these receptors in the mPFC [74–76]. The β-AR-induced increase in excitatory inputs, reduction in inhibitory inputs, and increased intrinsic excitability in S-neurons suggest that NE increases the signal-to-noise ratio [50–53,77]. This action of LC‒NE neurons is consistent with the modular projections and functions of the LC‒NE system. LC functions differ between projections to the PFC and BLA [23,60,61,69,78–80]. LC neurons also project to the amygdala and bed nucleus of the stria terminalis, and these neurons mediate anxiety or stress [31,81–83]. Whether the elevated BLA NE signals observed during memory retrieval may induce greater vigilance or stress remains to be examined [84]. Since LC neurons project to almost all brain regions and affect many cell types and brain functions, the extent to which they function in a modular manner and under which conditions they may exert a broader impact remain to be further clarified.

During US (footshock), elevated NE levels have been reported in both the BLA and PFC, among other brain regions [84–87]. This high NE level, especially in the BLA, has been shown to be required for the formation of threat memory and CS+-elicited freezing [88,89]. As NE is highly associated with vigilance, stress and anxiety, which, to some extent, are all related to threat responses [28–30,32,33,90], we propose that high dmPFC NE levels signal the importance of the CS and therefore are required for the initiation of behavior. In other words, the CS‒US association encoded in threat memory activates dmPFC neurons, the activity of which needs to be further boosted from high NE levels to reach the threshold for eliciting behavior. A low NE level either reflects the insignificance of US or is caused by the state of an organism (such as low excitability of LC neurons at certain brain functional states) and does not support the transition to behavior. Notably, the impact of the dmPFC NE is limited to the expression of conditioned behaviors but not memory itself. This is evidenced by the selective impacts of activated β-ARs on the spiking of S-neurons but not T-neurons. This finding also highlights the potential therapeutic value of targeting such neuromodulators.

It remains unknown if encoding of the valence/significance of CS via the dmPFC NE level is quantitative in that higher NE levels are linearly related to higher freezing levels, or, more specifically, if NE levels are scalable to freezing levels [39]. To test the relationship between these two parameters quantitatively, they both need to be in the linear range. Unfortunately, this requirement was unlikely to be met in the current study. First, we measured NE fluorescence levels rather than absolute NE concentrations, while the latter is required for determining this relationship. A conversion to NE concentrations cannot be performed using the florescence level measured at a single wavelength [55,57]. Second, we do not know where the linear range of freezing levels is under our current experimental setting, and freezing levels show high variability between mice. Nonetheless, a qualitative conclusion can be reached in that a threshold NE level is required for freezing to occur, and reducing NE uptake extends the window during which a CS can elicit freezing. Therefore, a high dmPFC NE level is required but not sufficient to elicit freezing, as a CS is also required (Fig 7A and 7B). This observation is consistent with the idea that NE is a neuromodulator rather than a driver (such as glutamate).

Another interesting finding of our current study is the generalization of freezing to nonconditioned cues. This generalization requires brief activation of the dmPFC and LC neurons to boost responses in the LC neurons induced by CS− (Fig 8F). This generalization is distinct from that assessed using separate CS+ and CS− stimuli; instead, it is revealed using the CS+/CS− hybrid stimuli. The short enhancement of dmPFC glutamatergic inputs to LC neurons after short-term stimulation appears to underlie this generalization (Fig 8G and 8H). Rapid plasticity induced by sensory stimulation in adult LC neurons [91] and long-lasting plasticity induced by stress in developing LC neurons [92] have been demonstrated. The reduced discrimination between CS+ and CS− is reminiscent of previous findings wherein a generalized increase in the LC–NE system elevates network gain without discrimination between inputs [93,94], therefore rendering the targeted circuits more responsive to a wide range of stimuli. Notably, this window of generalization is short since it only exists when the NE level is sufficiently high (Fig 7A and 7B). This observation suggests that the activation of LC neurons and the resulting high NE levels put the brain in an alert/vigilant state, during which sensory cues of the same modality readily elicit behavior regardless of their association with US. This notion of a temporary vigilance/alertness state is consistent with the idea that LC–NE is associated with vigilance and anxiety [28,61,95].

Taken together, the roles of the LC–NE system in the transition from memory to behavior are 2-fold: it enhances the response to behaviorally important cues, and it engages an emotion-like process that keeps the brain temporarily in a state ready to respond rapidly once the relevant cues appear. This latter function is consistent with LC activation by a task involving emotional cues [96] and is worthy of further exploration.

### Summary

Our findings demonstrate that the transition from memory to behavior is an elaborate process that requires the integration of memory, sensory inputs (CS), and valence/internal states (mediated by NE and likely other modulators) for best fitting to the CS encountered and the bodily states at the time of memory retrieval. This fine-tuning during the transition stage maintains the memory fidelity and dynamic adaptation of the generated behavior.

## Supporting information

S1 Fig Expression sites and efficiency of Cre- and DIO- viruses. (A) (A_1_) Site of virus injections. (A_2_) Representative images showing the spatial localization of dmPFC neurons expressing mCherry (Cre^+^ neurons) or EGFP (hSyn^+^ neurons), with white arrowheads indicate neurons were positive for both mCherry and EGFP. (A_3_) Ratio of mCherry^+^ versus EGFP^+^ neurons. *N* = 18 sections/6 mice. (B) (B_1_) Site of virus injections. (B_2_) Representative images showing the spatial localization of dmPFC neurons expressing mCherry (Cre and DIO^+^ neurons) or EGFP (hSyn^+^ neurons), with white arrowheads indicate neurons positive for both mCherry and EGFP. (B_3_) Ratio of mCherry^+^ versus EGFP^+^ neurons. *N* = 18 sections/6 mice. Numerical data can be found in S2 Data.
(TIF)

S2 Fig Responses elicited by CS+ and CS− in the dmPFC and BLA neurons during threat memory retrieval. (A) Sustained responses elicited by CS+ and no clear changes in responses by CS− in the S-neurons. *n* = 9 units/5 mice. (B) Transient responses elicited by CS+ or CS− in the LA neurons. *n* = 23 units/8 mice. (C) (Left) The averaged voltage waveforms of all inhibitory neurons (IN) and pyramidal neurons (PN). Thick lines represent the average and thin lines the SEM. Scale bars, 50 µV and 250 µs. (Middle) Quantification of half-width of spikes in the INs and PNs. PNs, 13 units/5 mice; INs, 10 units/6 mice; two-tailed *t* test, *P* < 0.001. (Right) Peak to trough and half-width of spikes in all recorded units. Numerical data can be found in S2 Data.
(TIF)

S3 Fig Electrophysiological properties of T-neurons and S-neurons in PFC slices. (A) sEPSC amplitude in the dmPFC T-neurons and S-neurons. One-way RM ANOVA, *F* (2, 53) = 2.804, Bonferroni’s posttest, *P* > 0.05; *n* = 15 cells/4 mice (NC-T), 15 cells/4 mice (NC-S), 20 cells/4 mice (Cond-T), 24 cells/6 mice (Cond-S). (B) Amplitude of AP trough in the PFC T-neurons and S-neurons. One-way RM ANOVA, *F* (3, 46) = 2.734, *P* > 0.05, Bonferroni’s posttest; 12 cells/4 mice (NC-T), 10 cells/4 mice (NC-S), 12 cells/4 mice (Cond-T), 12 cells/4 mice (Cond-S). (C) AP threshold in the PFC T-neurons and S-neurons. One-way RM ANOVA, *F* (3, 54) = 2.415, *P* > 0.05, Bonferroni’s posttest; 12 cells/4 mice (NC-T), 10 cells/4 mice (NC-S), 12 cells/4 mice (Cond-T), 12 cells/4 mice (Cond-S). (D) AHP latency in the PFC T-neurons and S-neurons. One-way RM ANOVA, *F* (3, 41) = 3.199, *P* < 0.05, Bonferroni’s posttest; Cond-T versus Cond-S, *P* < 0.05; 12 cells/4 mice (NC-T), 10 cells/4 mice (NC-S), 12 cells/4 mice (Cond-T), 12 cells/4 mice (Cond-S). (E) AHP peak in the PFC T-neurons and S-neurons. One-way RM ANOVA, *F* (3, 41) = 1.969, *P* > 0.05, Bonferroni’s posttest; 12 cells/4 mice (NC-T), 10 cells/4 mice (NC-S), 12 cells/4 mice (Cond-T), 12 cells/6 mice (Cond-S). **(F)** AP peak to trough in T-neurons and S-neurons. One-way RM ANOVA, *F* (3, 41) = 11.84, *P* < 0.001, Bonferroni’s posttest; NC-T versus NC-S, *P* < 0.001; NC-S versus Cond- S, *P* < 0.05; 12 cells/4 mice (NC-T), 10 cells/4 mice (NC-S), 12 cells/4 mice (Cond-T), 11 cells/4 mice (Cond-S). Numerical data can be found in S2 Data.
(TIF)

S4 Fig Sample traces testing the effects of Isop on T-neurons in the PFC slices. a(A) Sample traces of sEPSCs recorded from the T-neurons before and after bath application of Isop. Scale bars, 50 pA and 1 s. (B) Sample traces of sIPSCs recorded in the T-neurons before and after bath application of Isop. Scale bars, 50 pA and 500 ms.
(TIF)

S5 Fig Responses in the dmPFC reported by NE sensors with medium affinity (encoded by rAAV-hSyn-NE2m virus), in response to 30 s CS+. Scale bars, 0.5% *ΔF*/*F* and 10 s. *N* = 6 mice.
(TIF)

S6 Fig Sites of virus injections and optical fiber recording (left), and plots of NE signals in the BLA neurons elicited by 30 s CS+ and 30 s CS− (right). Scale bars, 0.5% *ΔF*/*F* and 10 s. Insert: AUC of *ΔF*/*F* of NE signals in the BLA neurons. Two-tailed unpaired *t* test, 30 s CS+ versus 30 s CS−, *P* < 0.001. *N* = 4 mice per group.
(TIF)

S7 Fig Examination of potential projections of LC neuron terminals in the LA or BLA, using retrobeads injected in the LC. Scale bar, 200 μm.
(TIF)

S8 Fig Examination of reciprocal connections between dmPFC and LC neurons. (A) (Left) Diagram showing the virus injection sites. The dmPFC neurons receiving LA inputs were labeled with mCherry (rAAV1-CaMKII-Cre virus injected in LA and rAAV2/9-Ef1α-DIO-mCherry virus in dmPFC). The LC-projecting dmPFC neurons were labeled with CTB 488 (green, injected in LC). (Right) Numbers of neurons positive for both mCherry^+^ and CTB 488^+^ over mCherry^+^ neurons and numbers of neurons positive for both mCherry^+^ and CTB 488^+^ over CTB 488^+^ neurons. *N* = 5 mice. (B) Representative images showing the spatial localization of dmPFC neurons expressing mCherry (red) or CTB 488 (green), taken with 10× (top) and 20× (bottom) objectives. The majority of labelled neurons were present in layer II/III of dmPFC. Scale bars, 100 μm (10×) and 20 μm (20×). Numerical data can be found in S2 Data.
(TIF)

S9 Fig Sample trace of recording in the dmPFC-projecting LC neurons with opto-activation of dmPFC neurons axon terminals in LC slices. Scale bars, 10 pA and 10 ms.
(TIF)

S10 Fig (A) Heat map of Ca^2+^ responses in the LC neurons that receive PFC projections, elicited by 5 sets of CSs. *N* = 7 mice. (B) Heat map of Ca^2+^ responses in the LC neurons projecting to dmPFC, elicited by 5 sets of CSs. *N* = 7 mice.
(TIF)

S11 Fig (Left) Diagram for virus injections. (Middle and right) Testing the effect of chemogenetic inhibition of BLA-projecting dmPFC neurons on the dmPFC NE signals and freezing levels. Scale bars, 1% *ΔF*/*F* and 10 s. Two-tailed paired *t* test, CS+ (Saline) versus CS+ (hM4D), *P* < 0.001; CS+ (hM4D) versus CS+ (mCherry), *P* < 0.001. *N* = 5 mice, each group. Insert: AUC of *ΔF*/*F* of dmPFC NE signals. Numerical data can be found in S2 Data.
(TIF)

S12 Fig Heat maps of dmPFC NE signals (A, *N* = 5 mice), LC Ca^2+^ responses (B, *N* = 6 mice) and Ca^2+^ signals in LC TH^+^ neurons (C, *N* = 7 mice), elicited by 2 s CS+/28 s CS−. (TIF)

S13 Fig Sample image of viral expression and fiber placement. The corresponding experiments were shown (for example, 2D refers to the experiments shown in Fig 2D). Scale bar, 100 μm.
(TIF)

S1 Table All virus used with their strain names, intended functions, regions injected and titer used. (XLSX)

S2 Table All detected genes and differential genes. (XLS)

S3 Table Genes encoding channels, receptor and CAMs. (XLS)

S1 Data Numerical data related to Figs 1C, 1F, 1G, 2A, 2C, 2F, 2G, 2L, 4E, 4F, 4G, 5D, 7A, 7B, 7C, 7D, 7E, 7F, 7G, 7H, 8F, 8G, 8H. (XLSX)

S2 Data Numerical data related to S1, S2C, S3, S8A, S11 Figs. (XLSX)

## Abbreviations

BLA: basolateral amygdala
CAM: cell-adhesion molecule
CeA: central nucleus of the amygdala
LA: lateral amygdala
LC: locus coeruleus
dmPFC: dorsomedial prefrontal cortex
PAG: periaqueductal gray
RMPs: resting membrane potentials
TeA: temporal association cortex

## References

[1] Pavlov I. Conditional Reflexes: An Investigation of the Physiological Activity of the Cerebral Cortex. 1927. 10.5214/ans.0972-7531.1017309 PMC4116985 25205891

[2] Rescorla RA. Behavioral studies of Pavlovian conditioning. Annu Rev Neurosci. 1988;11:329–52. doi: 10.1146/annurev.ne.11.030188.001553 3284445

[3] Pearce JM, Bouton ME. Theories of associative learning in animals. Annu Rev Psychol. 2001;52:111–39. doi: 10.1146/annurev.psych.52.1.111 11148301

[4] Fanselow MS, Wassum KM. The origins and organization of vertebrate Pavlovian conditioning. Cold Spring Harb Perspect Biol. 2015;8(1):a021717. doi: 10.1101/cshperspect.a021717 26552417 PMC4691796

[5] LeDoux JE. Emotion circuits in the brain. Annu Rev Neurosci. 2000;23:155–84. doi: 10.1146/annurev.neuro.23.1.155 10845062

[6] Maren S, Quirk GJ. Neuronal signalling of fear memory. Nat Rev Neurosci. 2004;5(11):844–52. doi: 10.1038/nrn1535 15496862

[7] Herry C, Johansen JP. Encoding of fear learning and memory in distributed neuronal circuits. Nat Neurosci. 2014;17(12):1644–54. doi: 10.1038/nn.3869 25413091

[8] Tovote P, Fadok JP, Lüthi A. Neuronal circuits for fear and anxiety. Nat Rev Neurosci. 2015;16(6):317–31. doi: 10.1038/nrn3945 25991441

[9] Sotres-Bayon F, Quirk GJ. Prefrontal control of fear: more than just extinction. Curr Opin Neurobiol. 2010;20(2):231–5. doi: 10.1016/j.conb.2010.02.005 20303254 PMC2878722

[10] Duvarci S, Pare D. Amygdala microcircuits controlling learned fear. Neuron. 2014;82(5):966–80. doi: 10.1016/j.neuron.2014.04.042 24908482 PMC4103014

[11] Gilmartin MR, Balderston NL, Helmstetter FJ. Prefrontal cortical regulation of fear learning. Trends Neurosci. 2014;37(8):455–64. doi: 10.1016/j.tins.2014.05.004 24929864 PMC4119830

[12] Gilmartin MR, McEchron MD. Single neurons in the medial prefrontal cortex of the rat exhibit tonic and phasic coding during trace fear conditioning. Behav Neurosci. 2005;119(6):1496–510. doi: 10.1037/0735-7044.119.6.1496 16420154

[13] Courtin J, Chaudun F, Rozeske RR, Karalis N, Gonzalez-Campo C, Wurtz H, et al. Prefrontal parvalbumin interneurons shape neuronal activity to drive fear expression. Nature. 2014;505(7481):92–6. doi: 10.1038/nature12755 24256726

[14] Yan R, Wang T, Zhou Q. Elevated dopamine signaling from ventral tegmental area to prefrontal cortical parvalbumin neurons drives conditioned inhibition. Proc Natl Acad Sci U S A. 2019;116(26):13077–86. doi: 10.1073/pnas.1901902116 31182594 PMC6600914

[15] Yan R, Wang T, Ma X, Zhang X, Zheng R, Zhou Q. Prefrontal inhibition drives formation and dynamic expression of probabilistic Pavlovian fear conditioning. Cell Rep. 2021;36(6):109503. doi: 10.1016/j.celrep.2021.109503 34380026

[16] Burgos-Robles A, Vidal-Gonzalez I, Quirk GJ. Sustained conditioned responses in prelimbic prefrontal neurons are correlated with fear expression and extinction failure. J Neurosci. 2009;29(26):8474–82. doi: 10.1523/JNEUROSCI.0378-09.2009 19571138 PMC2733220

[17] Pendyam S, Bravo-Rivera C, Burgos-Robles A, Sotres-Bayon F, Quirk GJ, Nair SS. Fear signaling in the prelimbic-amygdala circuit: a computational modeling and recording study. J Neurophysiol. 2013;110(4):844–61. doi: 10.1152/jn.00961.2012 23699055 PMC3742978

[18] Quirk GJ, Repa C, LeDoux JE. Fear conditioning enhances short-latency auditory responses of lateral amygdala neurons: parallel recordings in the freely behaving rat. Neuron. 1995;15(5):1029–39. doi: 10.1016/0896-6273(95)90092-6 7576647

[19] Goosens KA, Maren S. NMDA receptors are essential for the acquisition, but not expression, of conditional fear and associative spike firing in the lateral amygdala. Eur J Neurosci. 2004;20(2):537–48. 15233763 10.1111/j.1460-9568.2004.03513.x

[20] Tovote P, Esposito MS, Botta P, Chaudun F, Fadok JP, Markovic M, et al. Midbrain circuits for defensive behaviour. Nature. 2016;534(7606):206–12. doi: 10.1038/nature17996 27279213

[21] Giustino TF, Maren S. Noradrenergic modulation of fear conditioning and extinction. Front Behav Neurosci. 2018;12:43. doi: 10.3389/fnbeh.2018.00043 29593511 PMC5859179

[22] Likhtik E, Johansen JP. Neuromodulation in circuits of aversive emotional learning. Nat Neurosci. 2019;22(10):1586–97. doi: 10.1038/s41593-019-0503-3 31551602

[23] Uematsu A, Tan BZ, Ycu EA, Cuevas JS, Koivumaa J, Junyent F, et al. Modular organization of the brainstem noradrenaline system coordinates opposing learning states. Nat Neurosci. 2017;20(11):1602–11. doi: 10.1038/nn.4642 28920933

[24] Tully K, Li Y, Tsvetkov E, Bolshakov VY. Norepinephrine enables the induction of associative long-term potentiation at thalamo-amygdala synapses. Proc Natl Acad Sci U S A. 2007;104(35):14146–50. doi: 10.1073/pnas.0704621104 17709755 PMC1955781

[25] Bush DEA, Caparosa EM, Gekker A, Ledoux J. Beta-adrenergic receptors in the lateral nucleus of the amygdala contribute to the acquisition but not the consolidation of auditory fear conditioning. Front Behav Neurosci. 2010;4:154. doi: 10.3389/fnbeh.2010.00154 21152344 PMC2998038

[26] Sara SJ, Bouret S. Orienting and reorienting: the locus coeruleus mediates cognition through arousal. Neuron. 2012;76(1):130–41. doi: 10.1016/j.neuron.2012.09.011 23040811

[27] Rodriguez-Romaguera J, Sotres-Bayon F, Mueller D, Quirk GJ. Systemic propranolol acts centrally to reduce conditioned fear in rats without impairing extinction. Biol Psychiatry. 2009;65(10):887–92. doi: 10.1016/j.biopsych.2009.01.009 19246030 PMC2695810

[28] Bouras NN, Mack NR, Gao WJ. Prefrontal modulation of anxiety through a lens of noradrenergic signaling. Front Syst Neurosci. 2023;17. 37139472 10.3389/fnsys.2023.1173326 PMC10149815

[29] Koob GF. Corticotropin-releasing factor, norepinephrine, and stress. Biol Psychiatry. 1999;46(9):1167–80. doi: 10.1016/s0006-3223(99)00164-x 10560023

[30] Morris LS, McCall JG, Charney DS, Murrough JW. The role of the locus coeruleus in the generation of pathological anxiety. Brain Neurosci Adv. 2020;4:2398212820930321. doi: 10.1177/2398212820930321 32954002 PMC7479871

[31] Roozendaal B, McEwen BS, Chattarji S. Stress, memory and the amygdala. Nat Rev Neurosci. 2009;10(6):423–33. doi: 10.1038/nrn2651 19469026

[32] Sullivan GM, Coplan JD, Kent JM, Gorman JM. The noradrenergic system in pathological anxiety: a focus on panic with relevance to generalized anxiety and phobias. Biol Psychiatry. 1999;46(9):1205–18. doi: 10.1016/s0006-3223(99)00246-2 10560026

[33] Zerbi V, Floriou-Servou A, Markicevic M, Vermeiren Y, Sturman O, Privitera M, et al. Rapid reconfiguration of the functional connectome after chemogenetic locus coeruleus activation. Neuron. 2019;103(4):702–718.e5. doi: 10.1016/j.neuron.2019.05.034 31227310

[34] Aston-Jones G, Chiang C, Alexinsky T. Discharge of noradrenergic locus coeruleus neurons in behaving rats and monkeys suggests a role in vigilance. Prog Brain Res. 1991;88:501–20. doi: 10.1016/s0079-6123(08)63830-3 1813931

[35] Berridge CW, Waterhouse BD. The locus coeruleus-noradrenergic system: modulation of behavioral state and state-dependent cognitive processes. Brain Res Brain Res Rev. 2003;42(1):33–84. doi: 10.1016/s0165-0173(03)00143-7 12668290

[36] Morilak DA, Barrera G, Echevarria DJ, Garcia AS, Hernandez A, Ma S, et al. Role of brain norepinephrine in the behavioral response to stress. Prog Neuropsychopharmacol Biol Psychiatry. 2005;29(8):1214–24. doi: 10.1016/j.pnpbp.2005.08.007 16226365

[37] Arnsten AFT. Stress signalling pathways that impair prefrontal cortex structure and function. Nat Rev Neurosci. 2009;10(6):410–22. doi: 10.1038/nrn2648 19455173 PMC2907136

[38] Sara SJ. The locus coeruleus and noradrenergic modulation of cognition. Nat Rev Neurosci. 2009;10(3):211–23. doi: 10.1038/nrn2573 19190638

[39] Anderson DJ, Adolphs R. A framework for studying emotions across species. Cell. 2014;157(1):187–200. doi: 10.1016/j.cell.2014.03.003 24679535 PMC4098837

[40] Tervo DGR, Hwang B-Y, Viswanathan S, Gaj T, Lavzin M, Ritola KD, et al. A designer AAV variant permits efficient retrograde access to projection neurons. Neuron. 2016;92(2):372–82. doi: 10.1016/j.neuron.2016.09.021 27720486 PMC5872824

[41] Lin R, Wang R, Yuan J, Feng Q, Zhou Y, Zeng S, et al. Cell-type-specific and projection-specific brain-wide reconstruction of single neurons. Nat Methods. 2018;15(12):1033–6. doi: 10.1038/s41592-018-0184-y 30455464

[42] Smits JF, van Essen H, Struyker-Boudier HA. Propranolol in conscious spontaneously hypertensive rats. I. Cardiovascular effects after subcutaneous and intracerebroventricular administration. Naunyn Schmiedebergs Arch Pharmacol. 1979;309(1):13–8. doi: 10.1007/BF00498751 522893

[43] Xia D, Zhang X, Deng D, Ma X, Masri S, Wang J, et al. Long-term enhancement of NMDA receptor function in inhibitory neurons preferentially modulates potassium channels and cell adhesion molecules. Front Pharmacol. 2022;12:796179. doi: 10.3389/fphar.2021.796179 35058780 PMC8764260

[44] Wang T, Yan R, Zhang X, Wang Z, Duan H, Wang Z, et al. Paraventricular thalamus dynamically modulates aversive memory via tuning prefrontal inhibitory circuitry. J Neurosci. 2023;43(20):3630–46. doi: 10.1523/JNEUROSCI.1028-22.2023 37068932 PMC10198459

[45] Paré D, Collins DR. Neuronal correlates of fear in the lateral amygdala: multiple extracellular recordings in conscious cats. J Neurosci. 2000;20(7):2701–10. doi: 10.1523/JNEUROSCI.20-07-02701.2000 10729351 PMC6772231

[46] McGarry LM, Carter AG. Inhibitory gating of basolateral amygdala inputs to the prefrontal cortex. J Neurosci. 2016;36(36):9391–406. doi: 10.1523/JNEUROSCI.0874-16.2016 27605614 PMC5013187

[47] Kwon J-T, Jhang J, Kim H-S, Lee S, Han J-H. Brain region-specific activity patterns after recent or remote memory retrieval of auditory conditioned fear. Learn Mem. 2012;19(10):487–94. doi: 10.1101/lm.025502.112 22993170

[48] Cambiaghi M, Grosso A, Renna A, Concina G, Sacchetti B. Acute administration of nicotine into the higher order auditory Te2 cortex specifically decreases the fear-related charge of remote emotional memories. Neuropharmacology. 2015;99:577–88. doi: 10.1016/j.neuropharm.2015.08.036 26319210 PMC4710760

[49] Cho J-H, Huang BS, Gray JM. RNA sequencing from neural ensembles activated during fear conditioning in the mouse temporal association cortex. Sci Rep. 2016;6:31753. doi: 10.1038/srep31753 27557751 PMC4997356

[50] Polack P-O, Friedman J, Golshani P. Cellular mechanisms of brain state-dependent gain modulation in visual cortex. Nat Neurosci. 2013;16(9):1331–9. doi: 10.1038/nn.3464 23872595 PMC3786578

[51] Schiemann J, Puggioni P, Dacre J, Pelko M, Domanski A, van Rossum MCW, et al. Cellular mechanisms underlying behavioral state-dependent bidirectional modulation of motor cortex output. Cell Rep. 2015;11(8):1319–30. doi: 10.1016/j.celrep.2015.04.042 25981037 PMC4451462

[52] Skelly MJ, Ariwodola OJ, Weiner JL. Fear conditioning selectively disrupts noradrenergic facilitation of GABAergic inhibition in the basolateral amygdala. Neuropharmacology. 2017;113(Pt A):231–40. doi: 10.1016/j.neuropharm.2016.10.003 27720769 PMC5148638

[53] Bacon TJ, Pickering AE, Mellor JR. Noradrenaline release from locus coeruleus terminals in the hippocampus enhances excitation-spike coupling in CA1 pyramidal neurons via β-adrenoceptors. Cereb Cortex. 2020;30(12):6135–51. doi: 10.1093/cercor/bhaa159 32607551 PMC7609922

[54] Feenstra MG, Vogel M, Botterblom MH, Joosten RN, de Bruin JP. Dopamine and noradrenaline efflux in the rat prefrontal cortex after classical aversive conditioning to an auditory cue. Eur J Neurosci. 2001;13(5):1051–4. doi: 10.1046/j.0953-816x.2001.01471.x 11264679

[55] Feng J, Zhang C, Lischinsky JE, Jing M, Zhou J, Wang H, et al. A genetically encoded fluorescent sensor for rapid and specific in vivo detection of norepinephrine. Neuron. 2019;102(4):745–61 e8. 30922875 10.1016/j.neuron.2019.02.037 PMC6533151

[56] Ji Y, Onwukwe C, Smith J, Laub H, Posa L, Keller A, et al. Noradrenergic input from nucleus of the solitary tract regulates parabrachial activity in mice. eNeuro. 2023;10(5). 37072175 10.1523/ENEURO.0412-22.2023 PMC10162360

[57] Feng J, Dong H, Lischinsky J, Zhou J, Deng F, Zhuang C, et al. Monitoring norepinephrine release in vivo using next-generation GRABNE sensors. Neuron. 2023;112(12):1930–42. 38547869 10.1016/j.neuron.2024.03.001 PMC11364517

[58] Florin-Lechner SM, Druhan JP, Aston-Jones G, Valentino RJ. Enhanced norepinephrine release in prefrontal cortex with burst stimulation of the locus coeruleus. Brain Res. 1996;742(1–2):89–97. doi: 10.1016/s0006-8993(96)00967-5 9117425

[59] Berridge CW, Abercrombie ED. Relationship between locus coeruleus discharge rates and rates of norepinephrine release within neocortex as assessed by in vivo microdialysis. Neuroscience. 1999;93(4):1263–70. doi: 10.1016/s0306-4522(99)00276-6 10501450

[60] Chandler DJ, Waterhouse BD, Gao W-J. New perspectives on catecholaminergic regulation of executive circuits: evidence for independent modulation of prefrontal functions by midbrain dopaminergic and noradrenergic neurons. Front Neural Circuits. 2014;8:53. doi: 10.3389/fncir.2014.00053 24904299 PMC4033238

[61] Poe GR, Foote S, Eschenko O, Johansen JP, Bouret S, Aston-Jones G, et al. Locus coeruleus: a new look at the blue spot. Nat Rev Neurosci. 2020;21(11):644–59. doi: 10.1038/s41583-020-0360-9 32943779 PMC8991985

[62] Breton-Provencher V, Drummond GT, Sur M. Locus coeruleus norepinephrine in learned behavior: anatomical modularity and spatiotemporal integration in targets. Front Neural Circuits. 2021;15. 34163331 10.3389/fncir.2021.638007 PMC8215268

[63] Ross JA, Van Bockstaele EJ. The locus coeruleus–norepinephrine system in stress and arousal: unraveling historical, current, and future perspectives. Front Psychiatry. 2021;11. 33584368 10.3389/fpsyt.2020.601519 PMC7873441

[64] Kwok CHT, Harding EK, Burma NE, Markovic T, Massaly N, van den Hoogen NJ, et al. Pannexin-1 channel inhibition alleviates opioid withdrawal in rodents by modulating locus coeruleus to spinal cord circuitry. Nat Commun. 2024;15(1):6264. doi: 10.1038/s41467-024-50657-7 39048565 PMC11269731

[65] Arnsten AF, Goldman-Rakic PS. Selective prefrontal cortical projections to the region of the locus coeruleus and raphe nuclei in the rhesus monkey. Brain Res. 1984;306(1–2):9–18. doi: 10.1016/0006-8993(84)90351-2 6466989

[66] Jodo E, Chiang C, Aston-Jones G. Potent excitatory influence of prefrontal cortex activity on noradrenergic locus coeruleus neurons. Neuroscience. 1998;83(1):63–79. doi: 10.1016/s0306-4522(97)00372-2 9466399

[67] Barcomb K, Olah SS, Kennedy MJ, Ford CP. Properties and modulation of excitatory inputs to the locus coeruleus. J Physiol. 2022;600(22):4897–916. doi: 10.1113/JP283605 36156249 PMC9669264

[68] Hallock HL, Adiraju SS, Miranda-Barrientos J, McInerney JM, Oh S, DeBrosse AC, et al. Electrophysiological correlates of attention in the locus coeruleus-prelimbic cortex circuit during the rodent continuous performance test. Neuropsychopharmacology. 2023;49(3):521–31. doi: 10.1038/s41386-023-01692-3 37563281 PMC10789747

[69] Chandler DJ, Jensen P, McCall JG, Pickering AE, Schwarz LA, Totah NK. Redefining noradrenergic neuromodulation of behavior: impacts of a modular locus coeruleus architecture. J Neurosci. 2019;39(42):8239–49. doi: 10.1523/JNEUROSCI.1164-19.2019 31619493 PMC6794927

[70] Jodo E, Aston-Jones G. Activation of locus coeruleus by prefrontal cortex is mediated by excitatory amino acid inputs. Brain Res. 1997;768(1–2):327–32. doi: 10.1016/s0006-8993(97)00703-8 9369332

[71] Devoto P, Flore G, Saba P, Fà M, Gessa GL. Stimulation of the locus coeruleus elicits noradrenaline and dopamine release in the medial prefrontal and parietal cortex. J Neurochem. 2005;92(2):368–74. doi: 10.1111/j.1471-4159.2004.02866.x 15663484

[72] Chandler DJ, Gao W-J, Waterhouse BD. Heterogeneous organization of the locus coeruleus projections to prefrontal and motor cortices. Proc Natl Acad Sci U S A. 2014;111(18):6816–21. doi: 10.1073/pnas.1320827111 24753596 PMC4020069

[73] Kroes MCW, Tona K-D, den Ouden HEM, Vogel S, van Wingen GA, Fernández G. How administration of the beta-blocker propranolol before extinction can prevent the return of fear. Neuropsychopharmacology. 2016;41(6):1569–78. doi: 10.1038/npp.2015.315 26462618 PMC4820039

[74] Rainbow TC, Parsons B, Wolfe BB. Quantitative autoradiography of beta 1- and beta 2-adrenergic receptors in rat brain. Proc Natl Acad Sci U S A. 1984;81(5):1585–9. doi: 10.1073/pnas.81.5.1585 6324206 PMC344882

[75] Booze RM, Crisostomo EA, Davis JN. Beta-adrenergic receptors in the hippocampal and retrohippocampal regions of rats and guinea pigs: autoradiographic and immunohistochemical studies. Synapse. 1993;13(3):206–14. doi: 10.1002/syn.890130303 8098879

[76] Nicholas AP, Pieribone VA, Hökfelt T. Cellular localization of messenger RNA for beta-1 and beta-2 adrenergic receptors in rat brain: an in situ hybridization study. Neuroscience. 1993;56(4):1023–39. doi: 10.1016/0306-4522(93)90148-9 8284033

[77] Ferry B, Roozendaal B, McGaugh JL. Involvement of alpha1-adrenoceptors in the basolateral amygdala in modulation of memory storage. Eur J Pharmacol. 1999;372(1):9–16. doi: 10.1016/s0014-2999(99)00169-7 10374709

[78] Schwarz LA, Miyamichi K, Gao XJ, Beier KT, Weissbourd B, DeLoach KE, et al. Viral-genetic tracing of the input–output organization of a central noradrenaline circuit. Nature. 2015;524(7563):88–92. doi: 10.1038/nature14600 26131933 PMC4587569

[79] Hirschberg S, Li Y, Randall A, Kremer EJ, Pickering AE. Functional dichotomy in spinal- vs prefrontal-projecting locus coeruleus modules splits descending noradrenergic analgesia from ascending aversion and anxiety in rats. Elife. 2017;6. 29027903 10.7554/eLife.29808 PMC5653237

[80] Plummer NW, Chandler DJ, Powell JM, Scappini EL, Waterhouse BD, Jensen P. An intersectional viral-genetic method for fluorescent tracing of axon collaterals reveals details of noradrenergic locus coeruleus structure. eNeuro. 2020;7(3): 10–20. doi: 10.1523/ENEURO.0010-20.2020 32354756 PMC7294462

[81] Davis M. The role of the amygdala in fear-potentiated startle: implications for animal models of anxiety. Trends Pharmacol Sci. 1992;13(1):35–41. doi: 10.1016/0165-6147(92)90014-w 1542936

[82] Walker DL, Miles LA, Davis M. Selective participation of the bed nucleus of the stria terminalis and CRF in sustained anxiety-like versus phasic fear-like responses. Prog Neuro-Psychopharmacol Biol Psychiatry. 2009;33(8):1291–308. doi: 10.1016/j.pnpbp.2009.06.022 19595731 PMC2783512

[83] Adhikari A. Distributed circuits underlying anxiety. Front Behav Neurosci. 2014;8:112. doi: 10.3389/fnbeh.2014.00112 24744710 PMC3978252

[84] Goldstein LE, Rasmusson AM, Bunney BS, Roth RH. Role of the amygdala in the coordination of behavioral, neuroendocrine, and prefrontal cortical monoamine responses to psychological stress in the rat. J Neurosci. 1996;16(15):4787–98. doi: 10.1523/JNEUROSCI.16-15-04787.1996 8764665 PMC6579011

[85] Buffalari DM, Grace AA. Noradrenergic modulation of basolateral amygdala neuronal activity: opposing influences of alpha-2 and beta receptor activation. J Neurosci. 2007;27(45):12358–66. doi: 10.1523/JNEUROSCI.2007-07.2007 17989300 PMC6673273

[86] Lipski WJ, Grace AA. Footshock-induced responses in ventral subiculum neurons are mediated by locus coeruleus noradrenergic afferents. Eur Neuropsychopharmacol. 2013;23(10):1320–8. doi: 10.1016/j.euroneuro.2012.10.007 23394871 PMC3718869

[87] Giustino TF, Ramanathan KR, Totty MS, Miles OW, Maren S. Locus coeruleus norepinephrine drives stress-induced increases in basolateral amygdala firing and impairs extinction learning. J Neurosci. 2020;40(4):907–16. doi: 10.1523/JNEUROSCI.1092-19.2019 31801809 PMC6975297

[88] Shen W, Chen S, Xiang Y, Yao Z, Chen Z, Wu X, et al. Astroglial adrenoreceptors modulate synaptic transmission and contextual fear memory formation in dentate gyrus. Neurochem Int. 2021;143:104942. doi: 10.1016/j.neuint.2020.104942 33340594

[89] Isingrini E, Guinaudie C, Perret L, Guma E, Gorgievski V, Blum ID, et al. Behavioral and transcriptomic changes following brain-specific loss of noradrenergic transmission. Biomolecules. 2023;13(3):511. doi: 10.3390/biom13030511 36979445 PMC10046559

[90] Van Bockstaele EJ, Valentino RJ. Neuropeptide regulation of the locus coeruleus and opiate-induced plasticity of stress responses. Adv Pharmacol. 2013;68:405–20. doi: 10.1016/B978-0-12-411512-5.00019-1 24054155 PMC4707951

[91] Martins ARO, Froemke RC. Coordinated forms of noradrenergic plasticity in the locus coeruleus and primary auditory cortex. Nat Neurosci. 2015;18(10):1483–92. doi: 10.1038/nn.4090 26301326 PMC4583810

[92] Borodovitsyna O, Joshi N, Chandler D. Persistent stress-induced neuroplastic changes in the locus coeruleus/norepinephrine system. Neural Plast. 2018;2018:1892570. doi: 10.1155/2018/1892570 30008741 PMC6020552

[93] Usher M, Cohen JD, Servan-Schreiber D, Rajkowski J, Aston-Jones G. The role of locus coeruleus in the regulation of cognitive performance. Science. 1999;283(5401):549–54. doi: 10.1126/science.283.5401.549 9915705

[94] Aston-Jones G, Cohen JD. An integrative theory of locus coeruleus–norepinephrine function: adaptive gain and optimal performance. Annu Rev Neurosci. 2005;28:403–50. doi: 10.1146/annurev.neuro.28.061604.135709 16022602

[95] Suárez-Pereira I, Llorca-Torralba M, Bravo L, Camarena-Delgado C, Soriano-Mas C, Berrocoso E. The role of the locus coeruleus in pain and associated stress-related disorders. Biol Psychiatry. 2022;91(9):786–97. doi: 10.1016/j.biopsych.2021.11.023 35164940

[96] Sterpenich V, D’Argembeau A, Desseilles M, Balteau E, Albouy G, Vandewalle G, et al. The locus ceruleus is involved in the successful retrieval of emotional memories in humans. J Neurosci. 2006;26(28):7416–23. doi: 10.1523/JNEUROSCI.1001-06.2006 16837589 PMC6674193

