# Supplementary figures and images for: The locus coeruleus influences behavior by coordinating effective integration of fear memories and sensory input
Source: PLoS Biol. 2025 Jul 14;23(7):e3003272. doi: 10.1371/journal.pbio.3003272 (PMC12273993; doi:10.1371/journal.pbio.3003272)

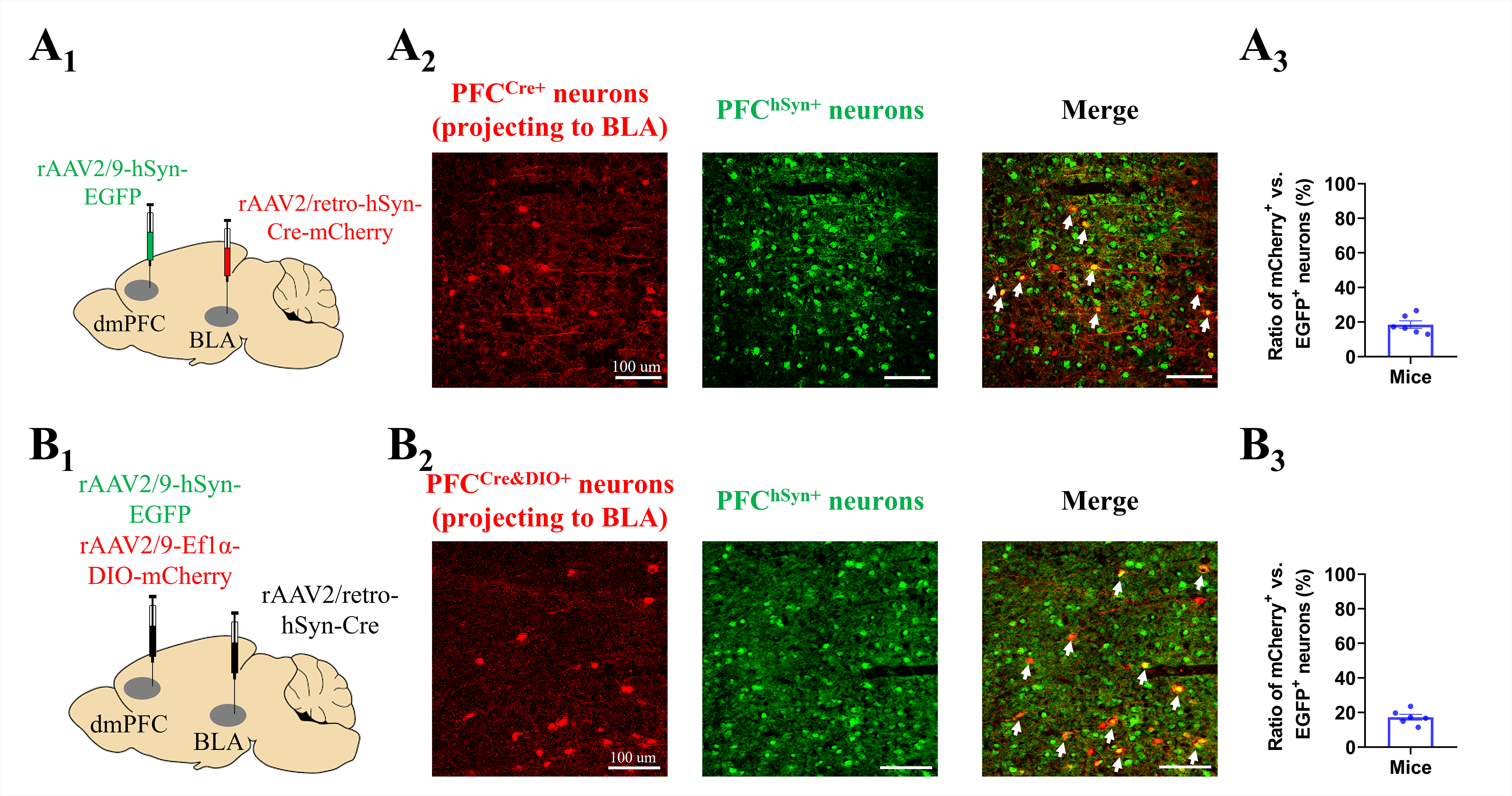

Supplement: S1 Fig — (A) (A1) Site of virus injections. (A2) Representative images showing the spatial localization of dmPFC neurons expressing mCherry (Cre+ neurons) or EGFP (hSyn+ neurons), with white arrowheads indicate neurons were positive for both mCherry and EGFP. (A3) Ratio of mCherry+ versus EGFP+ neurons. N = 18 sections/6 mice. (B) (B1) Site of virus injections. (B2) Representative images showing the spatial localization of dmPFC neurons expressing mCherry (Cre and DIO+ neurons) or EGFP (hSyn+ neurons), with white arrowheads indicate neurons positive for both mCherry and EGFP. (B3) Ratio of mCherry+ versus EGFP+ neurons. N = 18 sections/6 mice. Numerical data can be found in S2 Data. (TIF) [file pbio.3003272.s001.tif]

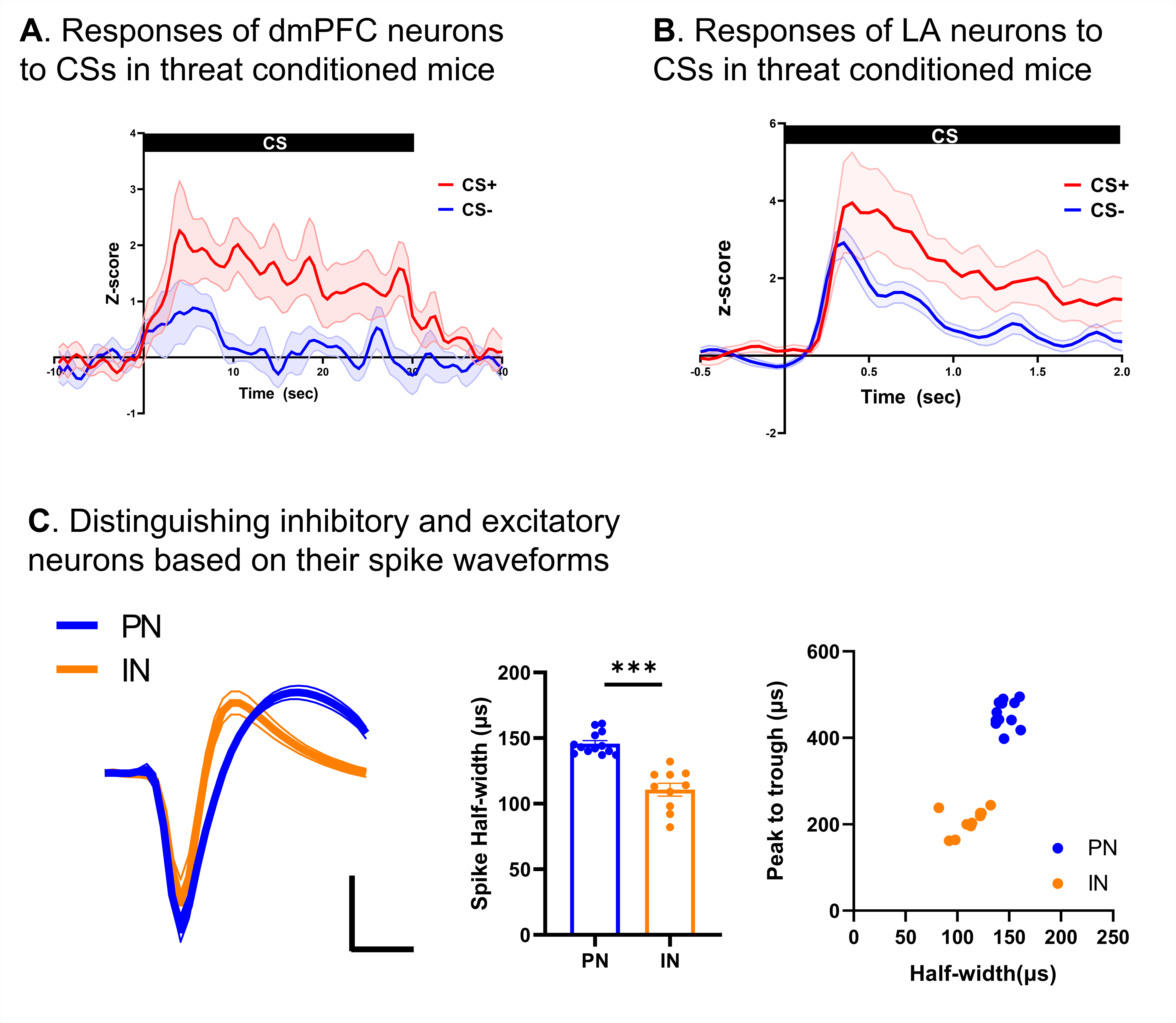

Supplement: S2 Fig — (A) Sustained responses elicited by CS+ and no clear changes in responses by CS− in the S-neurons. n = 9 units/5 mice. (B) Transient responses elicited by CS+ or CS− in the LA neurons. n = 23 units/8 mice. (C) (Left) The averaged voltage waveforms of all inhibitory neurons (IN) and pyramidal neurons (PN). Thick lines represent the average and thin lines the SEM. Scale bars, 50 µV and 250 µs. (Middle) Quantification of half-width of spikes in the INs and PNs. PNs, 13 units/5 mice; INs, 10 units/6 mice; two-tailed t test, P < 0.001. (Right) Peak to trough and half-width of spikes in all recorded units. Numerical data can be found in S2 Data. (TIF) [file pbio.3003272.s002.tif]

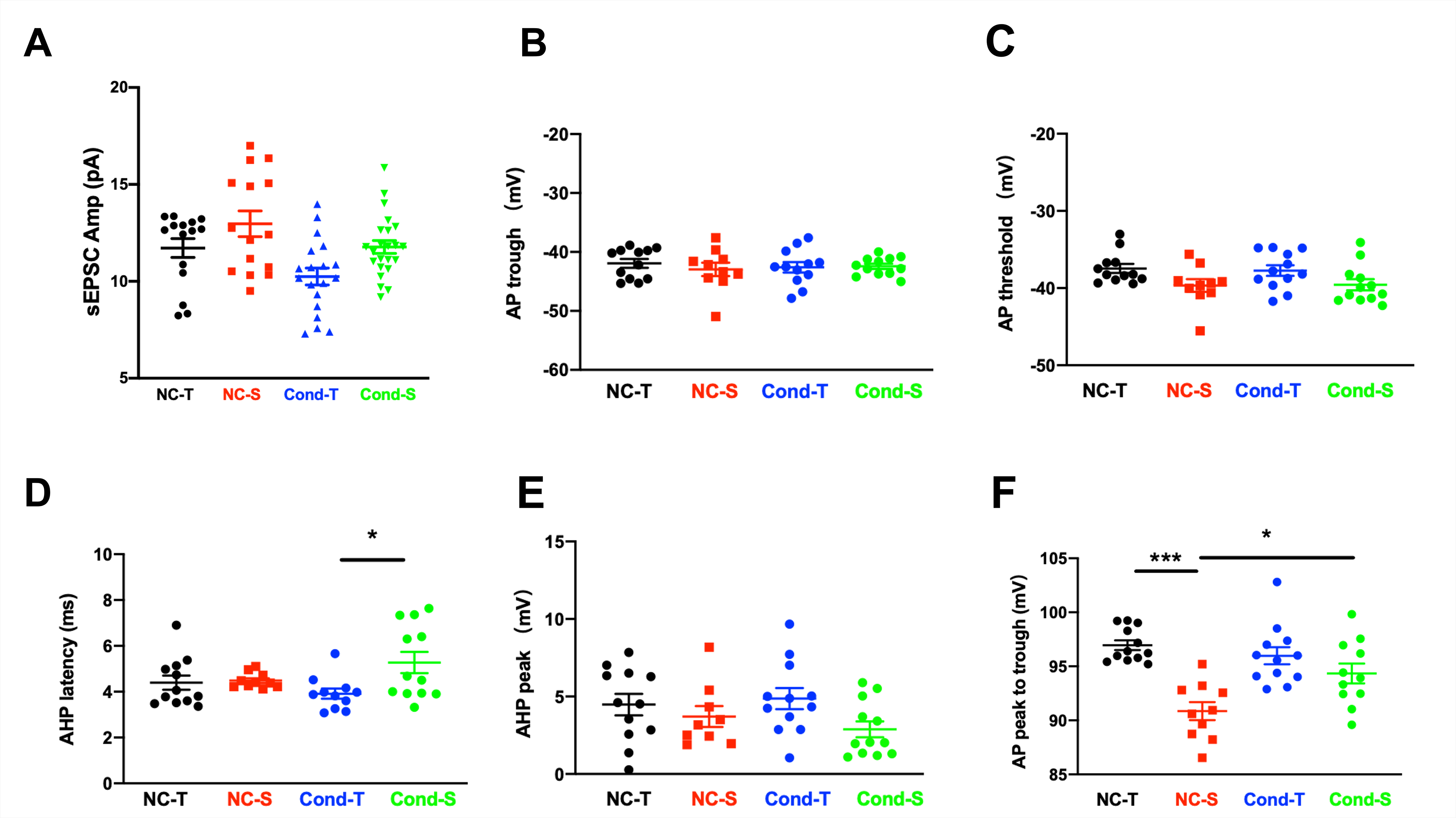

Supplement: S3 Fig — (A) sEPSC amplitude in the dmPFC T-neurons and S-neurons. One-way RM ANOVA, F (2, 53) = 2.804, Bonferroni’s posttest, P > 0.05; n = 15 cells/4 mice (NC-T), 15 cells/4 mice (NC-S), 20 cells/4 mice (Cond-T), 24 cells/6 mice (Cond-S). (B) Amplitude of AP trough in the PFC T-neurons and S-neurons. One-way RM ANOVA, F (3, 46) = 2.734, P > 0.05, Bonferroni’s posttest; 12 cells/4 mice (NC-T), 10 cells/4 mice (NC-S), 12 cells/4 mice (Cond-T), 12 cells/4 mice (Cond-S). (C) AP threshold in the PFC T-neurons and S-neurons. One-way RM ANOVA, F (3, 54) = 2.415, P > 0.05, Bonferroni’s posttest; 12 cells/4 mice (NC-T), 10 cells/4 mice (NC-S), 12 cells/4 mice (Cond-T), 12 cells/4 mice (Cond-S). (D) AHP latency in the PFC T-neurons and S-neurons. One-way RM ANOVA, F (3, 41) = 3.199, P < 0.05, Bonferroni’s posttest; Cond-T versus Cond-S, P < 0.05; 12 cells/4 mice (NC-T), 10 cells/4 mice (NC-S), 12 cells/4 mice (Cond-T), 12 cells/4 mice (Cond-S). (E) AHP peak in the PFC T-neurons and S-neurons. One-way RM ANOVA, F (3, 41) = 1.969, P > 0.05, Bonferroni’s posttest; 12 cells/4 mice (NC-T), 10 cells/4 mice (NC-S), 12 cells/4 mice (Cond-T), 12 cells/6 mice (Cond-S). (F) AP peak to trough in T-neurons and S-neurons. One-way RM ANOVA, F (3, 41) = 11.84, P < 0.001, Bonferroni’s posttest; NC-T versus NC-S, P < 0.001; NC-S versus Cond- S, P < 0.05; 12 cells/4 mice (NC-T), 10 cells/4 mice (NC-S), 12 cells/4 mice (Cond-T), 11 cells/4 mice (Cond-S). Numerical data can be found in S2 Data. (TIF) [file pbio.3003272.s003.tif]

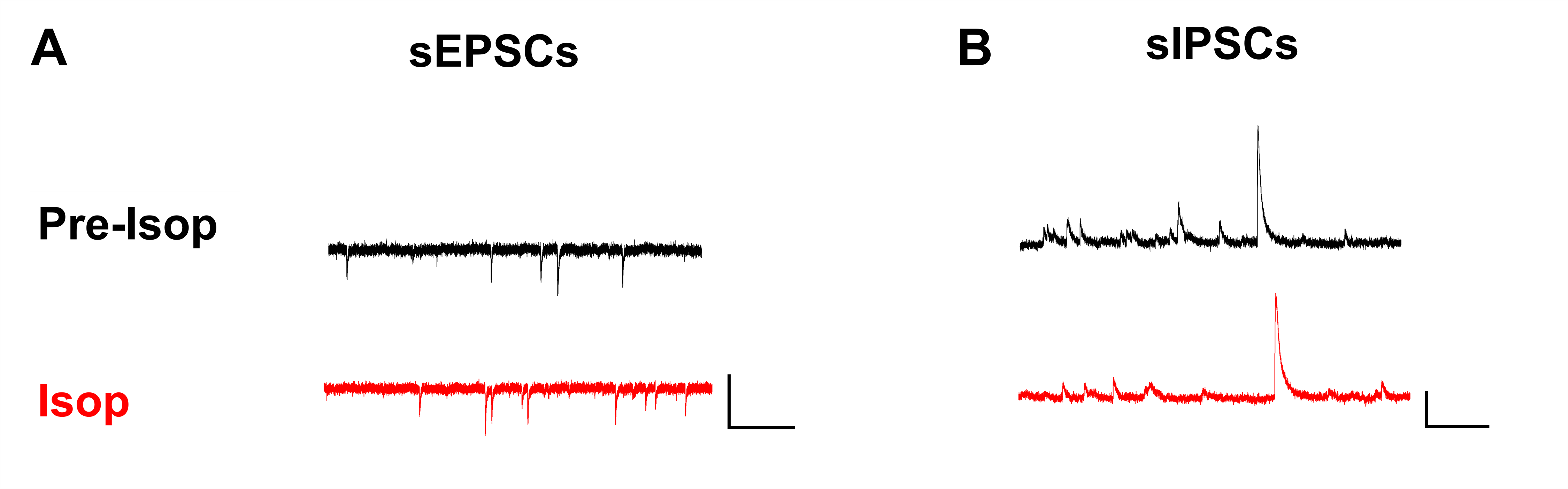

Supplement: S4 Fig — a(A) Sample traces of sEPSCs recorded from the T-neurons before and after bath application of Isop. Scale bars, 50 pA and 1 s. (B) Sample traces of sIPSCs recorded in the T-neurons before and after bath application of Isop. Scale bars, 50 pA and 500 ms. (TIF) [file pbio.3003272.s004.tif]

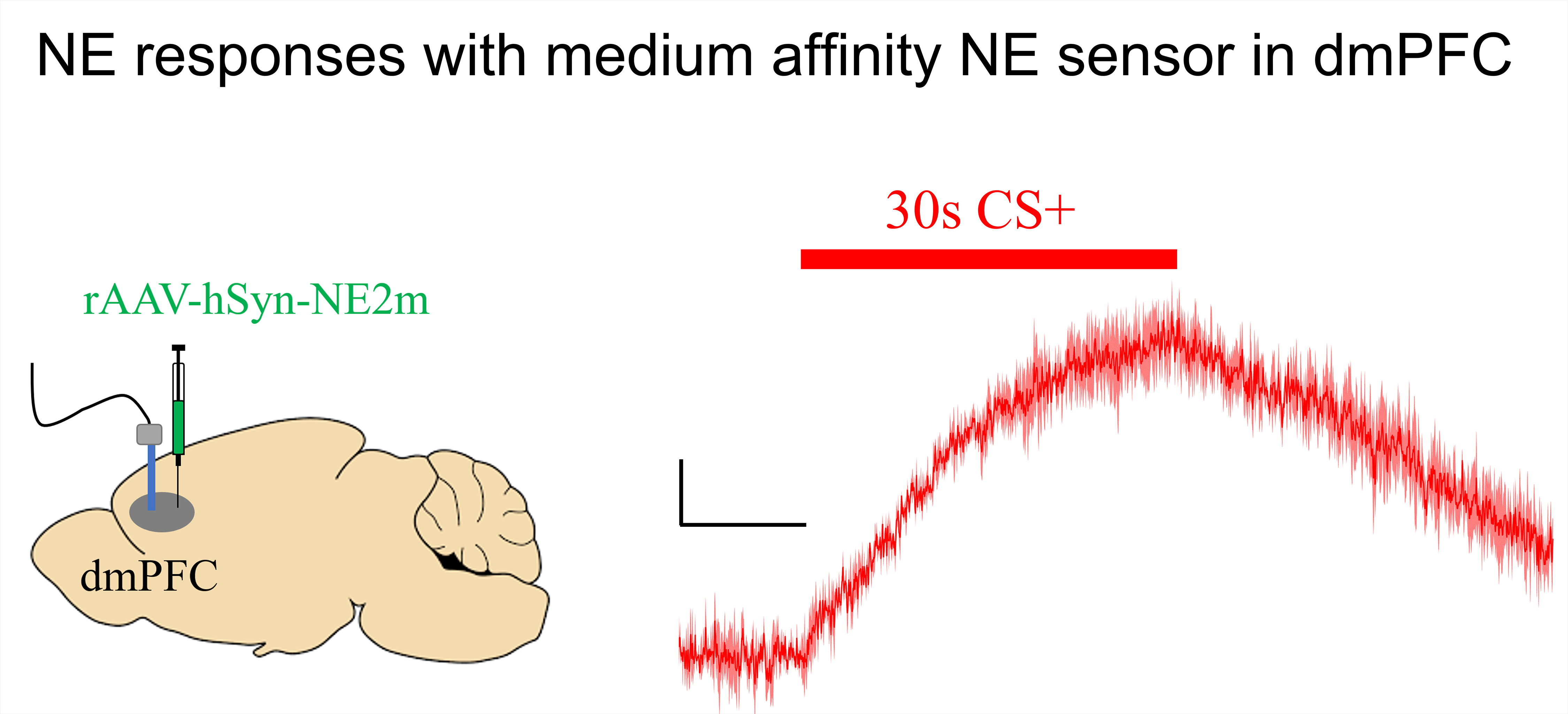

Supplement: S5 Fig — Scale bars, 0.5% ΔF/F and 10 s. N = 6 mice. (TIF) [file pbio.3003272.s005.tif]

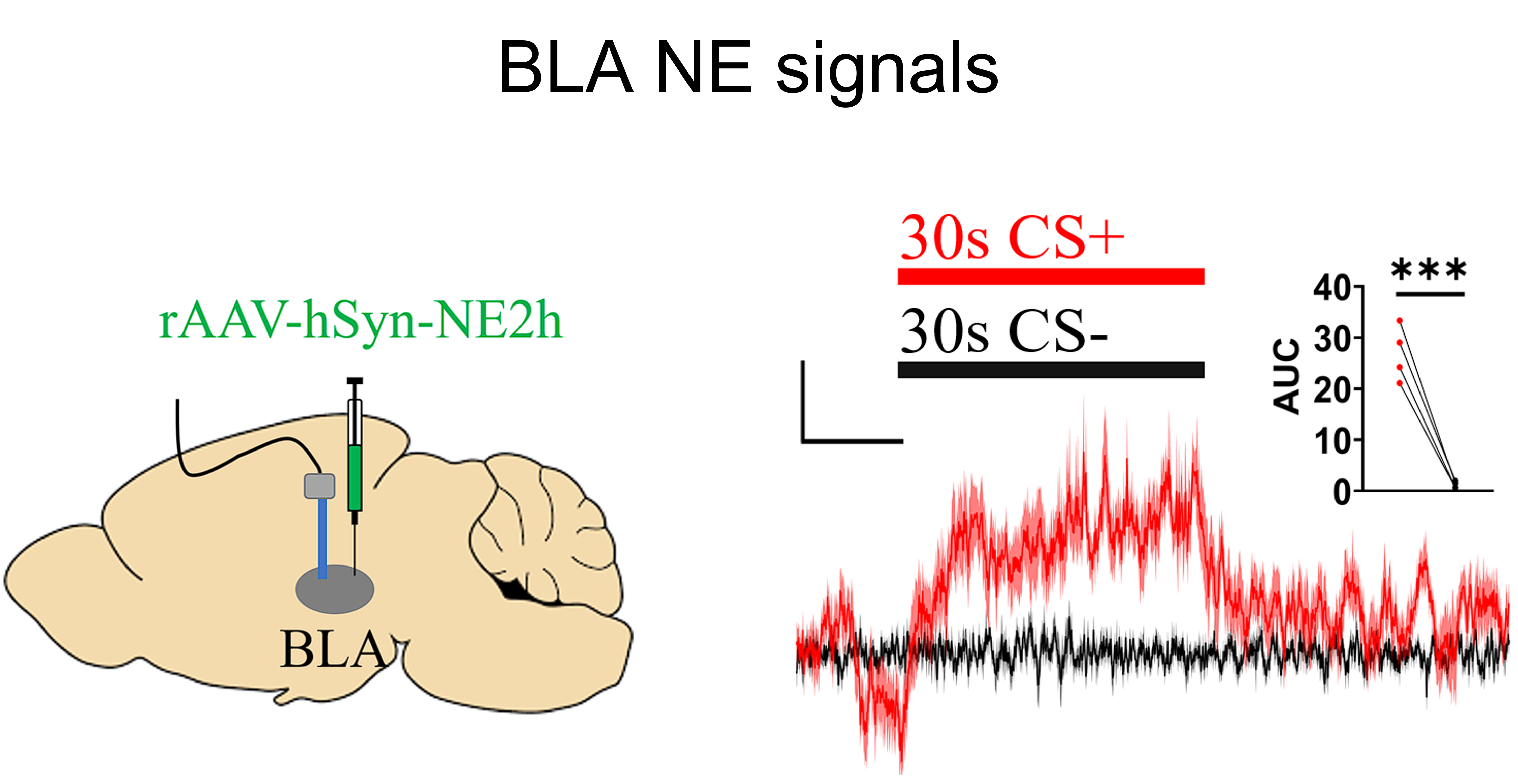

Supplement: S6 Fig — Scale bars, 0.5% ΔF/F and 10 s. Insert: AUC of ΔF/F of NE signals in the BLA neurons. Two-tailed unpaired t test, 30 s CS+ versus 30 s CS−, P < 0.001. N = 4 mice per group. (TIF) [file pbio.3003272.s006.tif]

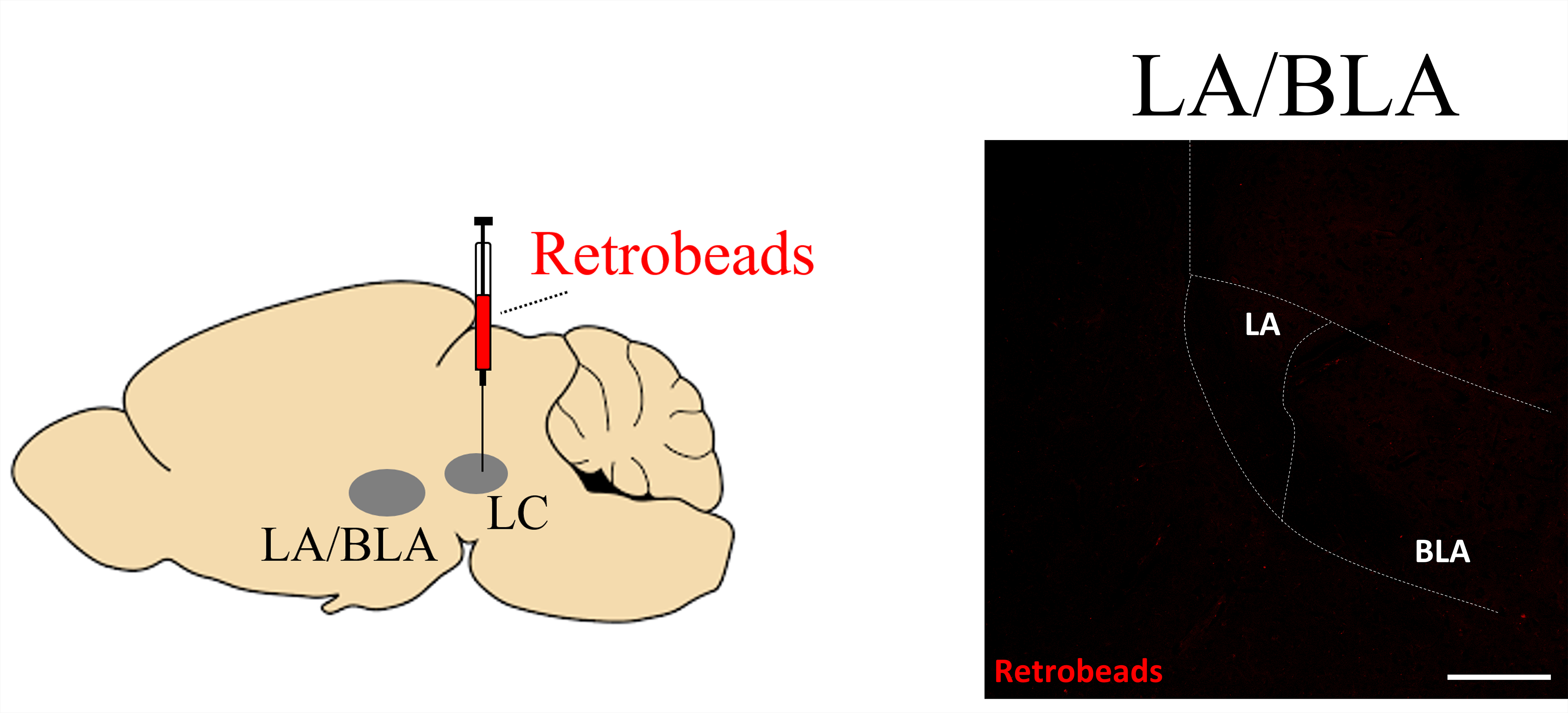

Supplement: S7 Fig — Scale bar, 200 μm. (TIF) [file pbio.3003272.s007.tif]

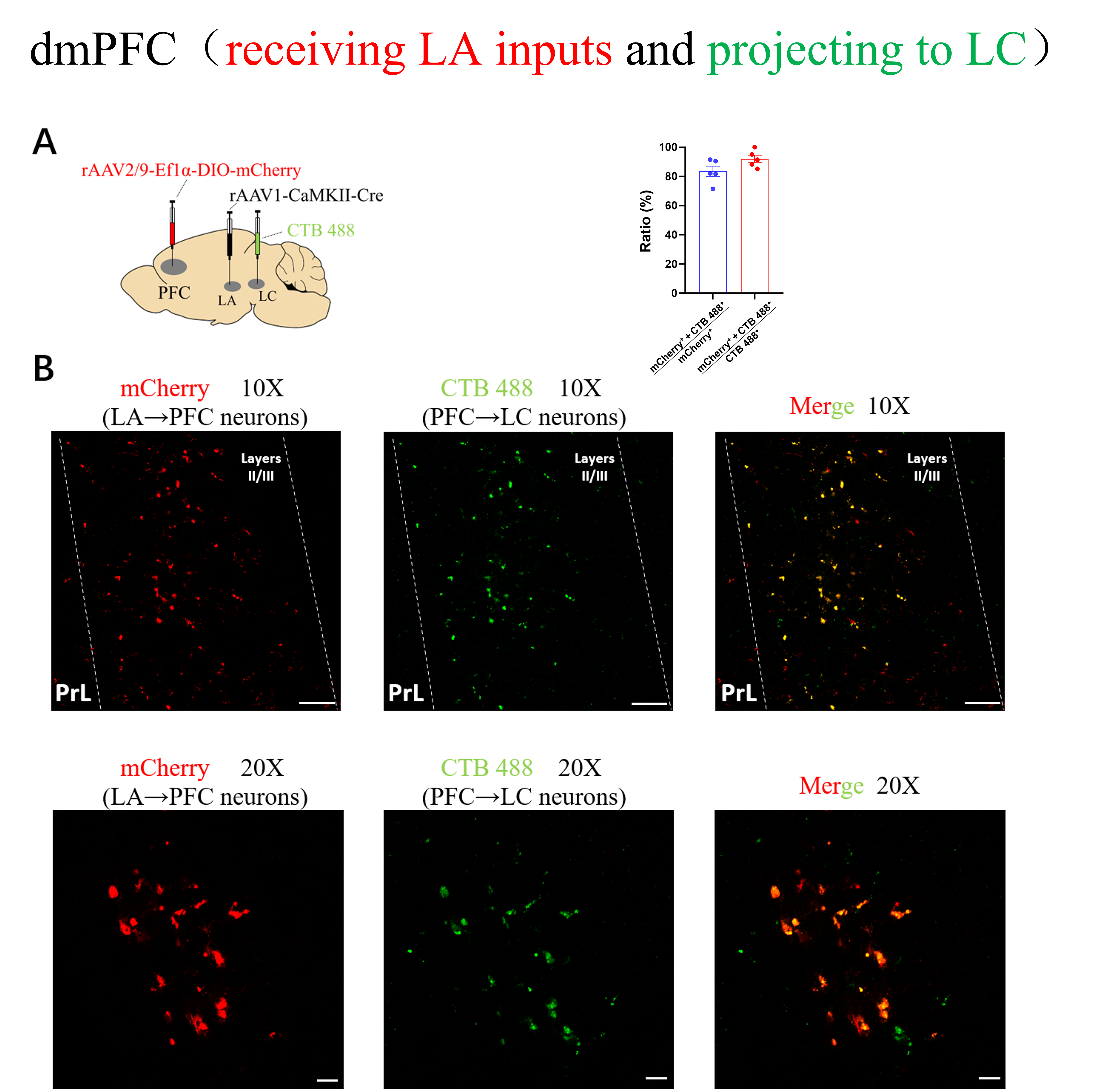

Supplement: S8 Fig — (A) (Left) Diagram showing the virus injection sites. The dmPFC neurons receiving LA inputs were labeled with mCherry (rAAV1-CaMKII-Cre virus injected in LA and rAAV2/9-Ef1α-DIO-mCherry virus in dmPFC). The LC-projecting dmPFC neurons were labeled with CTB 488 (green, injected in LC). (Right) Numbers of neurons positive for both mCherry+ and CTB 488+ over mCherry+ neurons and numbers of neurons positive for both mCherry+ and CTB 488+ over CTB 488+ neurons. N = 5 mice. (B) Representative images showing the spatial localization of dmPFC neurons expressing mCherry (red) or CTB 488 (green), taken with 10× (top) and 20× (bottom) objectives. The majority of labelled neurons were present in layer II/III of dmPFC. Scale bars, 100 μm (10×) and 20 μm (20×). Numerical data can be found in S2 Data. (TIF) [file pbio.3003272.s008.tif]

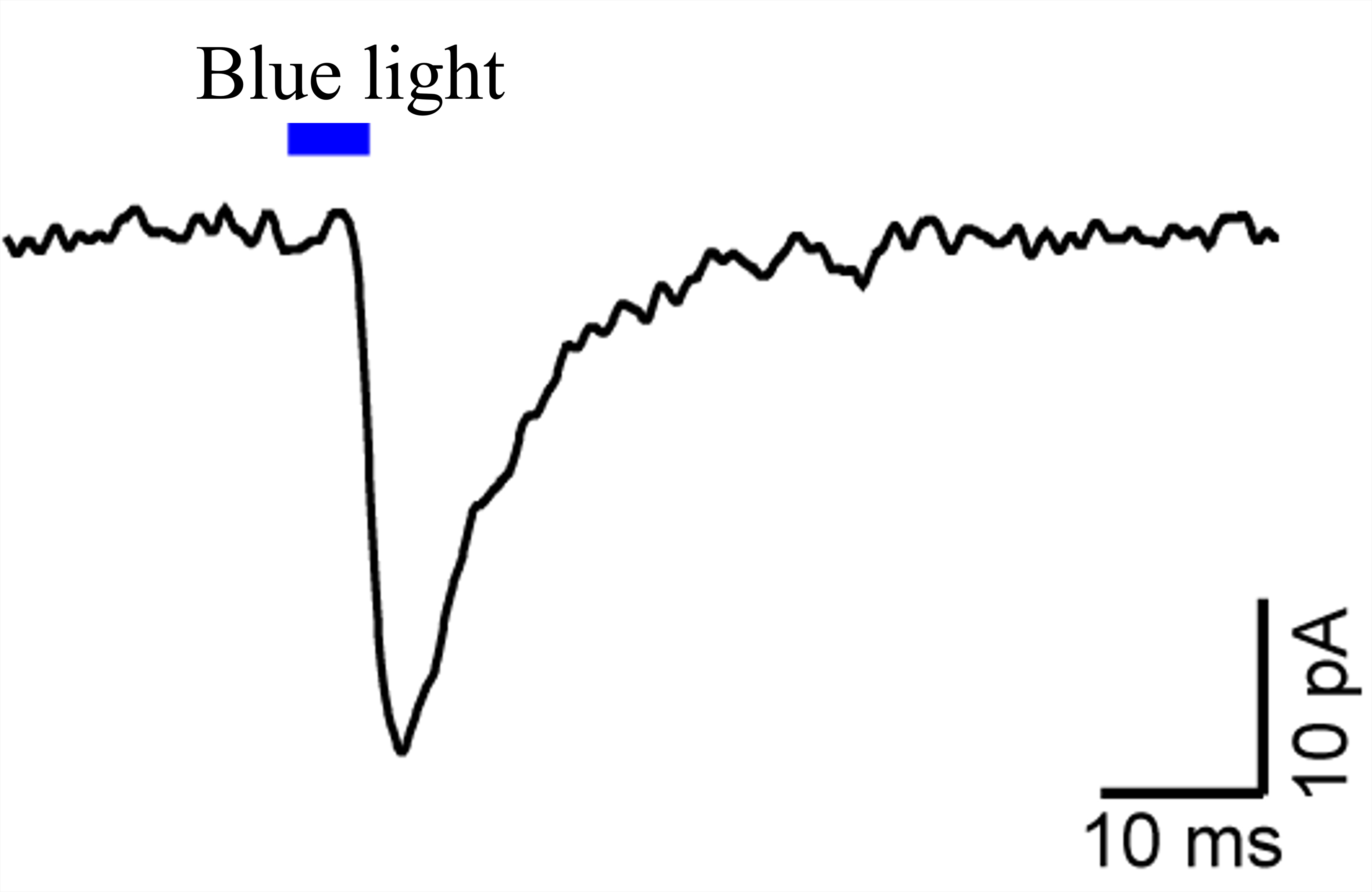

Supplement: S9 Fig — Scale bars, 10 pA and 10 ms. (TIF) [file pbio.3003272.s009.tif]

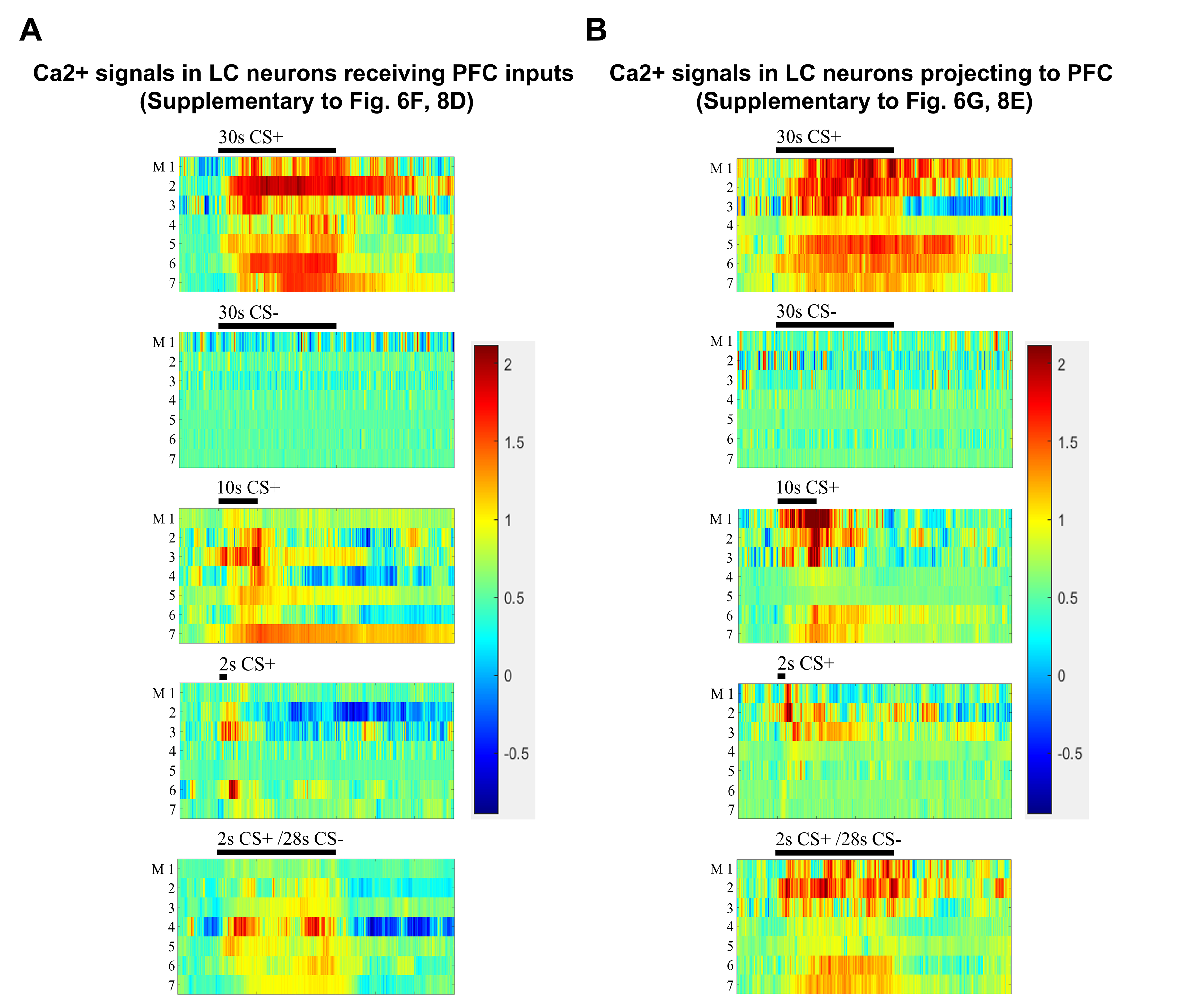

Supplement: S10 Fig — N = 7 mice. (B) Heat map of Ca2+ responses in the LC neurons projecting to dmPFC, elicited by 5 sets of CSs. N = 7 mice. (TIF) [file pbio.3003272.s010.tif]

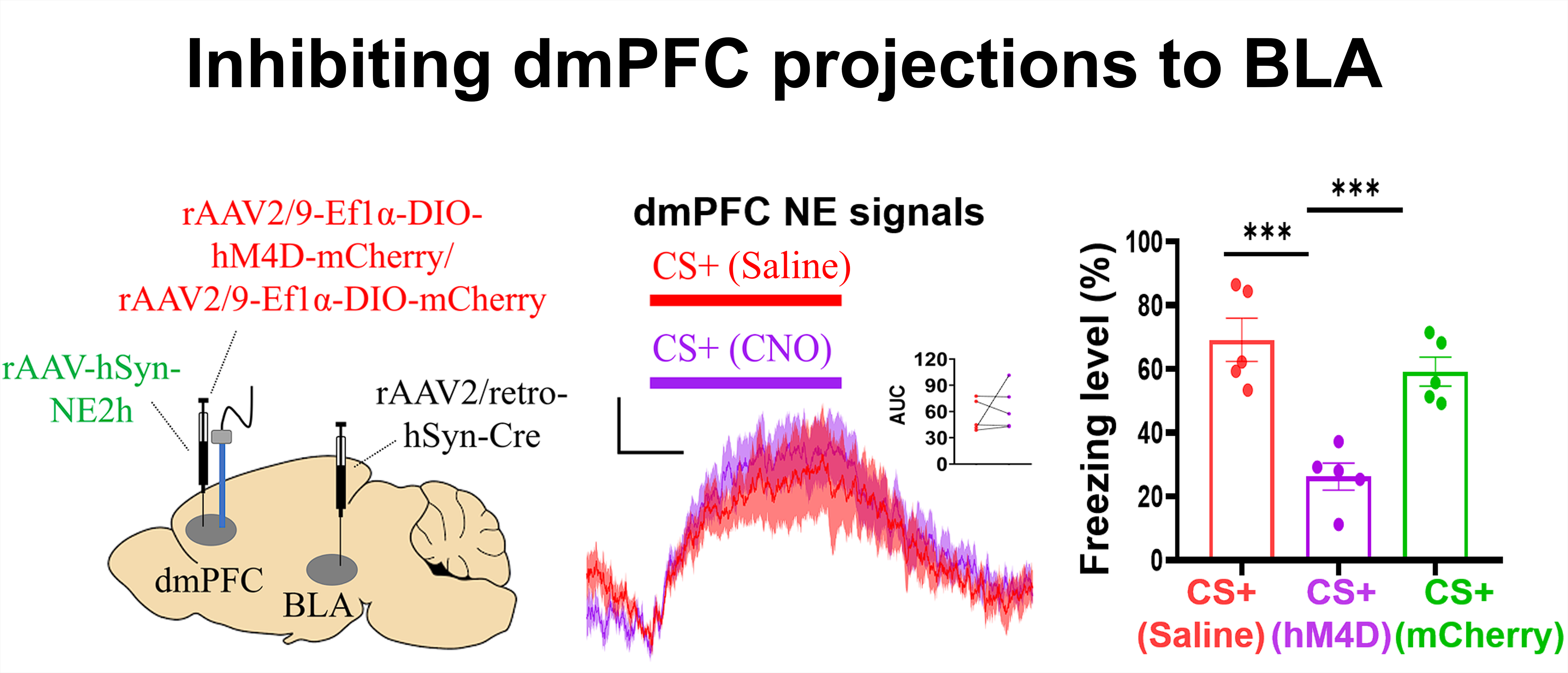

Supplement: S11 Fig — (Middle and right) Testing the effect of chemogenetic inhibition of BLA-projecting dmPFC neurons on the dmPFC NE signals and freezing levels. Scale bars, 1% ΔF/F and 10 s. Two-tailed paired t test, CS+ (Saline) versus CS+ (hM4D), P < 0.001; CS+ (hM4D) versus CS+ (mCherry), P < 0.001. N = 5 mice, each group. Insert: AUC of ΔF/F of dmPFC NE signals. Numerical data can be found in S2 Data. (TIF) [file pbio.3003272.s011.tif]

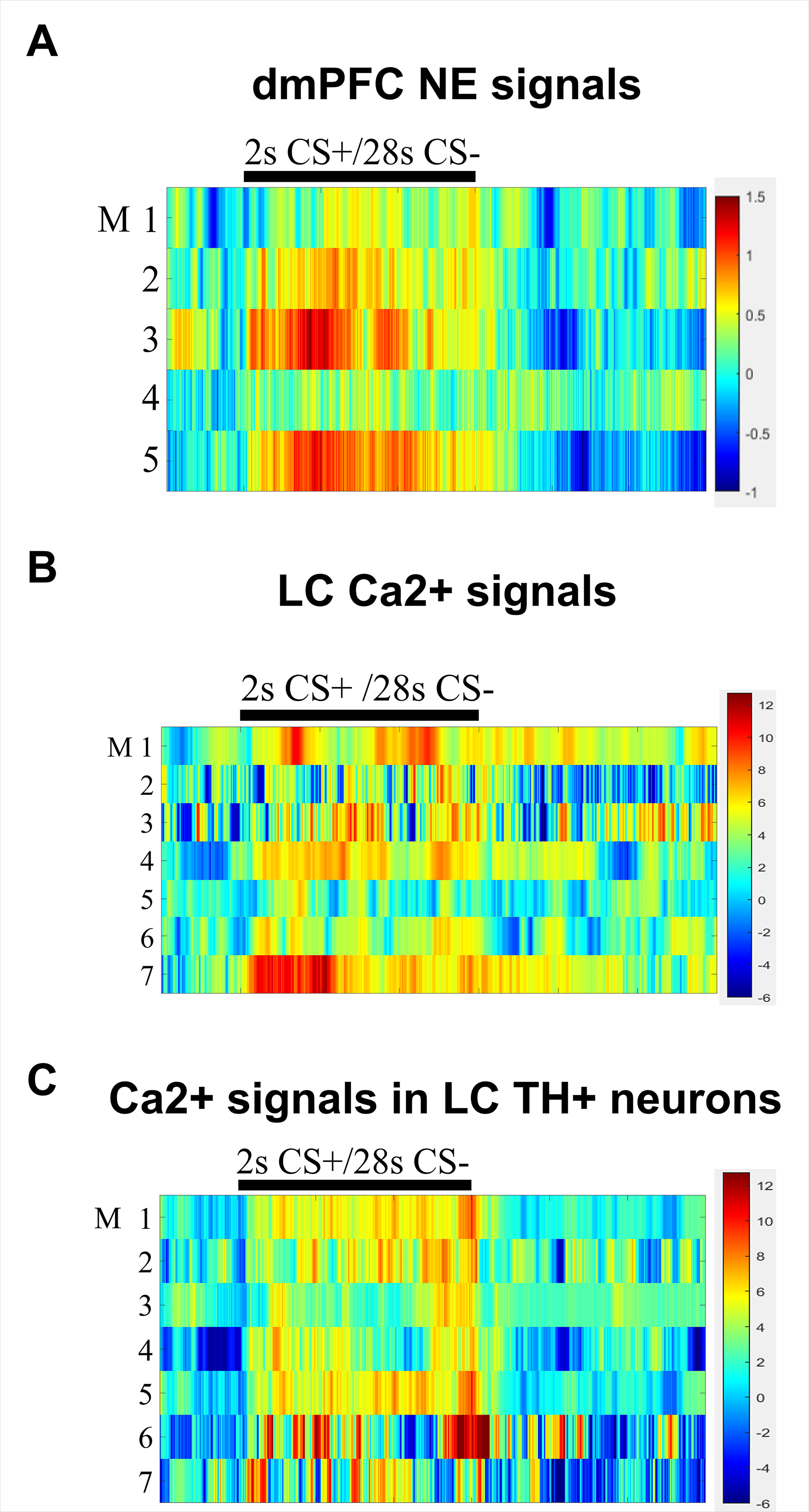

Supplement: S12 Fig — (TIF) [file pbio.3003272.s012.tif]

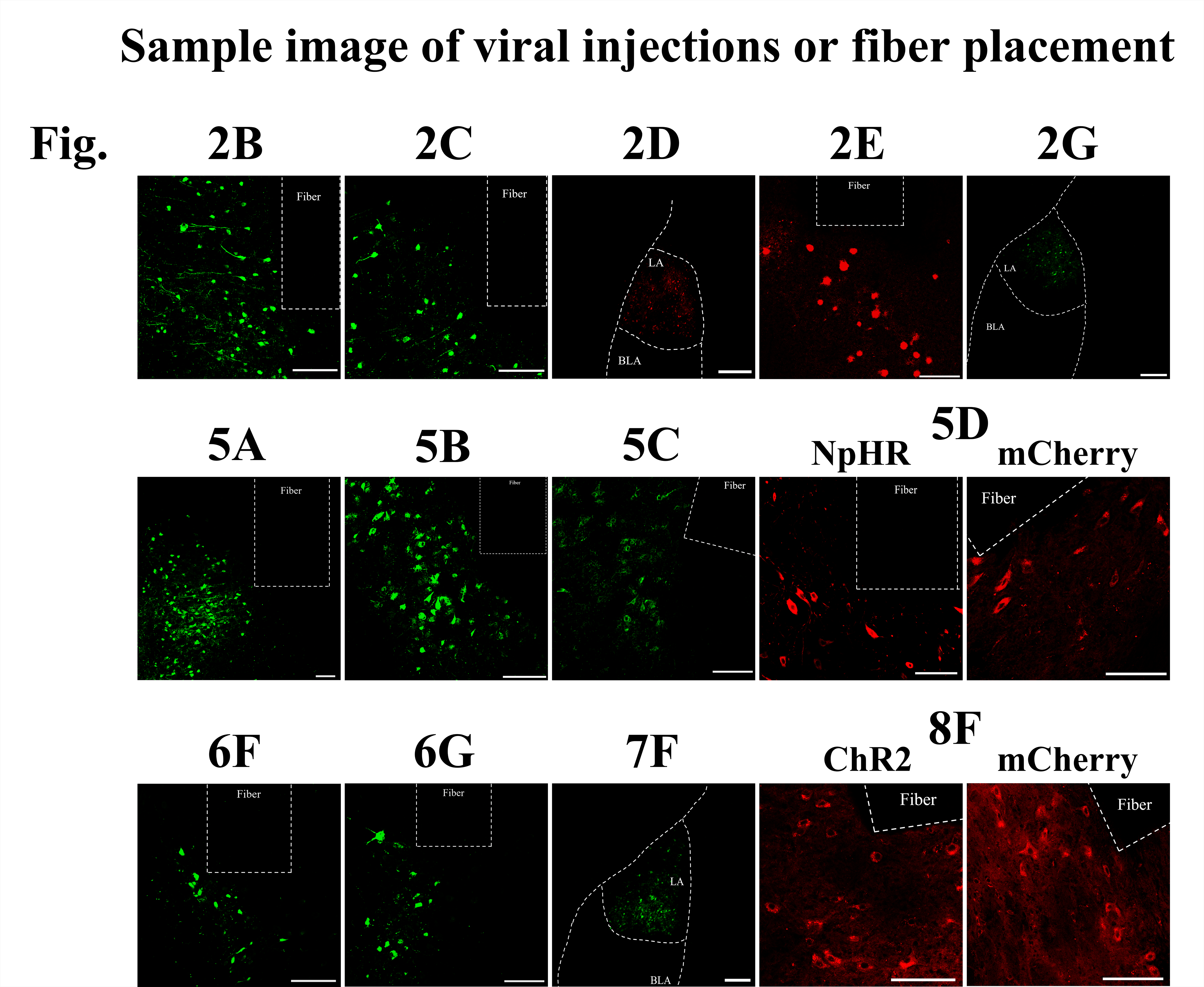

Supplement: S13 Fig — The corresponding experiments were shown (for example, 2D refers to the experiments shown in Fig 2D). Scale bar, 100 μm. (TIF) [file pbio.3003272.s013.tif]
